# Exploring the role of different cell types on cortical folding in the developing human brain through computational modeling

**DOI:** 10.1038/s41598-024-75952-7

**Published:** 2024-10-30

**Authors:** Mohammad Saeed Zarzor, Qiang Ma, Median Almurey, Bernhard Kainz, Silvia Budday

**Affiliations:** 1 Institute of Continuum Mechanics and Biomechanics, Friedrich-Alexander-Universität Erlangen-Nürnberg, 91058 Erlangen, Germany; 2 Department of Computing, Imperial College London, London, SW7 2AZ UK; 3 Erlangen Graduate School in Advanced Optical Technologies, Friedrich-Alexander-Universität Erlangen-Nürnberg, 91052 Erlangen, Germany

**Keywords:** Cortical folding, Human brain development, Outer radial glial cells, Multi-field modeling, Coupled problems, Computational biology and bioinformatics, Cell biology

## Abstract

The human brain’s distinctive folding pattern has attracted the attention of researchers from different fields. Neuroscientists have provided insights into the role of four fundamental cell types crucial during embryonic development: radial glial cells, intermediate progenitor cells, outer radial glial cells, and neurons. Understanding the mechanisms by which these cell types influence the number of cortical neurons and the emerging cortical folding pattern necessitates accounting for the mechanical forces that drive the cortical folding process. Our research aims to explore the correlation between biological processes and mechanical forces through computational modeling. We introduce cell-density fields, characterized by a system of advection-diffusion equations, designed to replicate the characteristic behaviors of various cell types in the developing brain. Concurrently, we adopt the theory of finite growth to describe cortex expansion driven by increasing cell density. Our model serves as an adjustable tool for understanding how the behavior of individual cell types reflects normal and abnormal folding patterns. Through comparison with magnetic resonance images of the fetal brain, we explore the correlation between morphological changes and underlying cellular mechanisms. Moreover, our model sheds light on the spatiotemporal relationships among different cell types in the human brain and enables cellular deconvolution of histological sections.

## Introduction

 The human brain is the command center for motion, sensory experiences, communication, and reasoning. Its unique folding pattern, alongside the highest neuron-to-size density across species, and its ultra-soft mechanical properties, make it the arguably most complex organ in the human body^1,2^. The brain’s cognitive functions are intricately linked to two factors, the complexity of its folds and the density of neurons that populate its cortical layer. Indeed, these two factors are interconnected: an increase in neuronal count leads to more pronounced folding, while detailed folding patterns shorten the distance between neurons, which improves their communication. This interaction is essential for the advanced functions and capabilities of the brain^3^.

In recent decades, research questions such as how neurons develop during the initial stages of human brain development to achieve such vast numbers in a relatively short period of time, the way in which an increase in a neuronal count contributes to the expansion of the cortex, and the mechanism of cortical folding, have been in the center of attention. The majority of earlier studies had concentrated on genetic and biological aspects to address these questions. However, this approach turned out to be insufficient; rather, a consideration of various aspects of human brain development, including mechanical effects is required. For instance, mechanical forces play a crucial role for the cortical buckling process that leads to the formation of folds^4–7^.

To understand the role of these forces on the resulting folding pattern, numerous computational models have been created in recent years^4,8,9^. However, a purely mechanical viewpoint is also insufficient. It is crucial to consider both mechanics and the biological processes. In the past few years, several computational models have thus been developed to account for the interaction between macroscopic mechanical forces and microscopic cellular processes^10–12^. More recently, these have also considered the cell division behavior in different proliferating zones of the human brain and their effect on the emerging cortical folding pattern^13,14^. These models have shown good agreement with behaviors observed in the actual human brain. However, they do not account for the different cell types present in the brain during development. Therefore, they fails to explain how neurons form in the early stages or how this influences the brain’s folding patterns.

To address this limitation, we present an extension of our previous model^13,14^ to additionally account for the behaviors of different cell types during human brain development, integrating their roles with mechanical forces and structural changes. Our approach aims to provide a more comprehensive understanding of brain development, particularly in terms of neuronal formation and the emergence of normal and abnormal cortical folds.

## Materials and methods

### Biological background

**Fig. 1:**
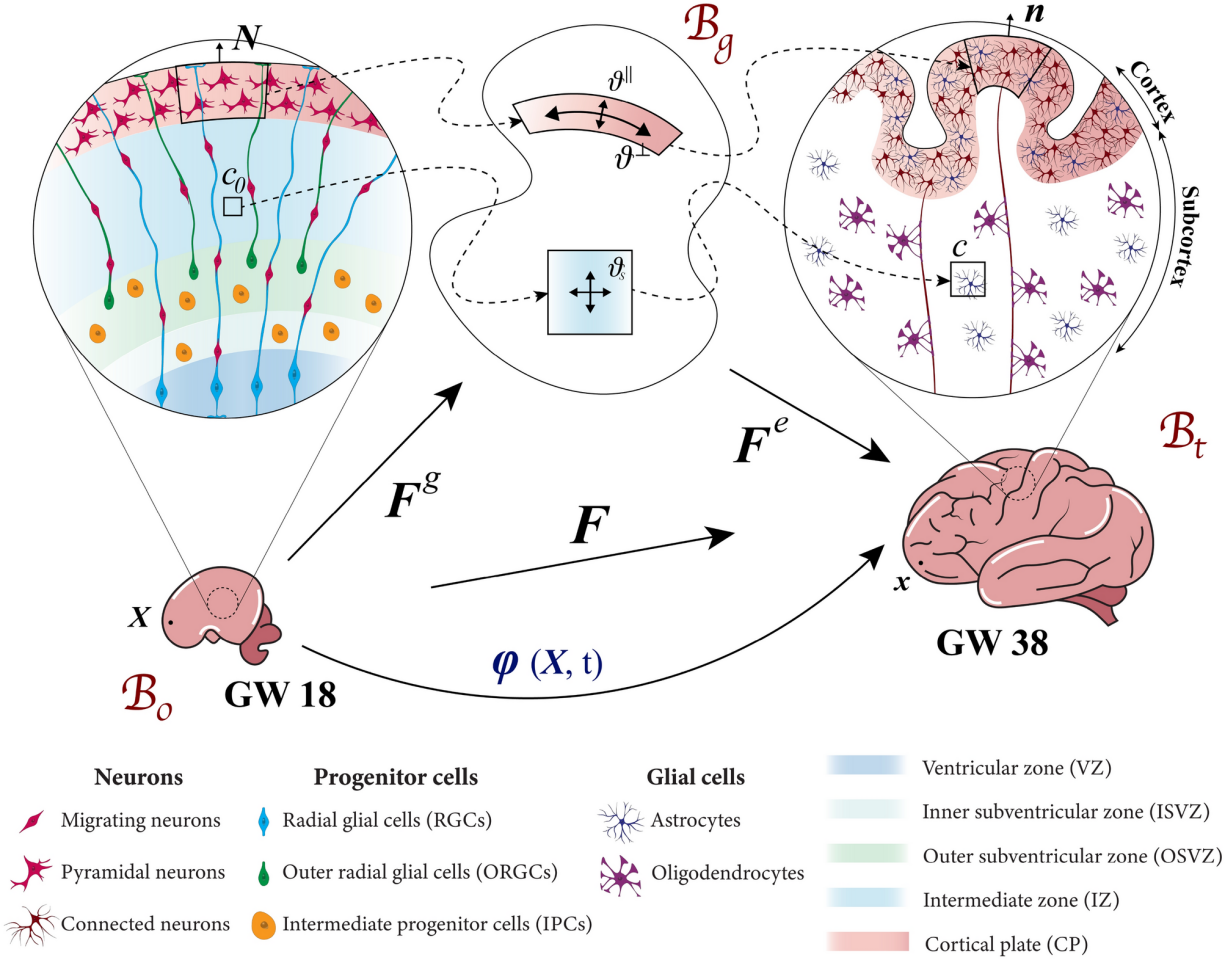
Kinematics of human brain growth model between gestational weeks (GW) 18 and 38. The deformation map ${\varvec{{x}}}=\varvec{\varphi }({\varvec{{X}}}, t)$ maps the material point ${\varvec{{X}}}$ from the stress-free reference configuration $\mathcal {B}_\text{0}$ with outward pointing normal vector ${\varvec{{N}}}$ to the spatial configuration $\mathcal {B}_\text{t}$ with outward pointing normal vector $\varvec{n}$ at a specific time *t*. Inserting the intermediate growth configuration $\mathcal {B}_\text{g}$ leads to a multiplicative decomposition of the deformation gradient ${\varvec{{F}}} = \varvec{\nabla }_{{\varvec{{X}}}} \varvec{\varphi }$ into an elastic component, $\varvec{F}^\text{e}$, and a growth component, ${\varvec{{F}}}^\text{g}$. The Jacobian $J= \text {det} \, {\varvec{{F}}}$ maps the cell density *c* from the reference to spatial configuration, i.e., $c_0 = Jc$. The cellular processes on the microscopic scale drive the cortical $\vartheta ^\parallel$,$\vartheta ^\bot$, and subcortical $\vartheta _{\text{s}}$ growth multipliers in $\mathcal {B}_\text{g}$.

During human brain development, four fundamental cell types play an essential role, radial glial cells (RGCs), intermediate progenitor cells (IPCs), Outer radial glial cells (ORGCs), and neurons, as illustrated in Fig. 1, left. At the very beginning of neurogenesis, the original stem cells, which are called neuroepithelial cells, transform into RGCs in the brain’s ventricular zone around the cerebral ventricles^15^. Between gestational weeks 5 and 7, the RGCs amplify their number through symmetric division that leads to a significant increase in both the thickness and surface area of the ventricular zone (VZ)^16–18^. Thereafter, the newly generated RGCs switch from symmetric to asymmetric division and become the initial source of IPCs^19^. The IPCs migrate to populate the subventricular zone (SVZ), located above the ventricular zone (VZ). In the SVZ, IPCs undergo multiple rounds of division before they ultimately produce cortical neurons^20,21^. Importantly, apart from the limited quantity of neurons generated by RGCs during the initial stages of neurogenesis, IPCs serve as the exclusive source of cortical neurons^3,22^. In gyrencephalic species, especially in humans, by around gestational week 11, RGCs undergo a transition from generating IPCs to generating ORGCs^23–25^. The latter exhibit a distinctive division behavior, swiftly translocating upon generation to the outer layer of the SVZ, forming a distinct zone known as the outer subventricular zone (OSVZ). This unique division behavior is reffed to as ’mitotic somal translocation’ (MST)^24,26,27^. As a result of this behavior, the boundary of the OSVZ expands outward in radial direction over time, which increases its capacity to produce new cells^21^. During gestational weeks 11 to 24, ORGCs undergo asymmetric division to produce IPCs, making ORGCs the second source of these cells^22^. The abundance of IPCs in gyrencephalic species plays a crucial role in doubling the neurons generated. Consequently, these species exhibit a significantly greater number of cortical neurons compared to lissencephalic species^24^. The newly generated neurons migrate towards the cortical layer, arranging themselves in a sequence from inside to outside to form the six-layered cortex^28^. According to the radial unit hypothesis, the fibers of both RGCs and ORGCs form a scaffold for neuronal migration^29^. Therefore, both types of radial glial cells function as central units for both cell generation and the organization of neuronal migration. After gestational week 23, brain folds start to emerge as the outer brain surface significantly expands circumferentially^5,30^, as indicated in Fig. 1, right. Intriguingly, this expansion of the brain’s surface coincides with the development of neuronal connectivity^31^.

### Computational model

To numerically study the role of the different cell types mentioned in the previous section on the resulting folding patterns during human brain development, we develop a multi-field computational model coupling growth and mechanical deformation with a cell-density field. The cell density field is characterized by a system of advection-diffusion equations, each formulated to mimic the distinct behavior of a specific cell type. The mechanical deformation is coupled to the cell density field as growth is controlled by the cell density. In the subsequent sections, we present the fundamental equations that outline this coupled problem. Initially, we provide a broad overview of the kinematics of finite growth, the mechanical balance, and the constitutive equations. Following this, we delve into the specifics of the cell-density problem. Next, we introduce the discretization process in space and time, and finally, we discuss the selection and calibration of model parameters.

#### Kinematics

In our approach, we use the principles of nonlinear continuum mechanics, enriched with concepts from the theory of finite growth. On the macroscopic (tissue) scale, we define the deformation map ${\varvec{{x}}} = \varvec{\varphi } ({\varvec{{X}}}, t)$ to depict the transition of a material point from the brain’s initial (reference) state, $\mathcal {B}_{0} \subset \mathbb {R}^3$, to its spatial (deformed) state, $\mathcal {B}_\text{t} \subset \mathbb {R}^3$, at a subsequent time $t \in \mathbb {R}_{+}$ during its development, with ${\varvec{{x}}} \in \mathcal {B}_\text{t}$ and ${\varvec{{X}}} \in \mathcal {B}_0$. Additionally, we introduce the deformation gradient, ${\varvec{{F}}} = \varvec{\nabla }_{{\varvec{{X}}}} \varvec{\varphi }$, a derivative of the deformation map with respect to the position vector of the reference point, to transform a line element from its original to its new position. The determinant of the deformation gradient, the Jacobian $J= \text {det} \, {\varvec{{F}}}$, is used to measure the resulting local volumetric change, as illustrated in Fig. 1. Consistent with the theory of finite growth, an intermediate (stress-free) configuration, $\mathcal {B}_\text{g}$, is inserted between the brain’s two primary configurations. Consequently, the deformation gradient is multiplicatively decomposed into an elastic component, ${\varvec{{F}}}^\text{e}$, and a growth component, ${\varvec{{F}}}^\text{g}$^32,33^, such that

1 $$\varvec{F} = \varvec{F}^{{\text{e}}} \cdot \varvec{F}^{{\text{g}}} .$$

Likewise, the Jacobian is split into $J = J^\text{e} \cdot J^\text{g}$, where $J^\text{e} = \text {det} \, {\varvec{{F}}}^\text{e}$, and $J^\text{g} = \text {det} \, {\varvec{{F}}}^\text{g}$. It should be highlighted that the elastic deformation tensor captures the brain’s purely elastic response to both external and internal forces, which preserves the continuity of the tissue. In contrast, the growth tensor specifies the extent and direction of free expansion led by the cell-density field. Notably, the elastic deformation is reversible, whereas the growth part leads to an irreversible change in the system.

To represent the microscopic (cell) scale, we introduce the independent scalar field of material cell density, $c_0({\varvec{{X}}})$, which represents the total number of cells per unit volume of the brain in its reference state. The spatial cell density, $c({\varvec{{x}}},t)$, defined for a specific time *t* in the deformed configuration, is linked to the material cell density through the Jacobian as $c_0 = Jc$.

#### Mechanics problem

For the mechanics problem, we solve the balance of linear momentum in the absence of external forces, which is represented in the spatial configuration as,

2 $$\begin{aligned} \text {div} \,\varvec{\sigma } = {\textbf {0}}, \end{aligned}$$

where $\text {div}$ is the divergence operator with respect to spatial position ${\varvec{{x}}}$, and $\varvec{\sigma }$ is the Cauchy stress tensor. The stress tensor is derived from the elastic deformation tensor since only elastic deformations contribute to stress within the material, i.e., $\varvec{\sigma } = \varvec{\sigma }({\varvec{{F}}}^\text{e})$.

We assume that the response of brain tissue during development is isotropic and hyperelastic, as viscous effects, notable at higher strain rates, diminish in significance over the developmental stages that span months of gestation. Moreover, within the specific regions of interest, i.e., the subcortex and cortex, the tissues have shown isotropic properties in experiments^2,34^. Mathematically, we introduce the strain energy function $\psi _0$ in the spatial configuration $\mathcal {B}_{0}$, that is corresponding to function $\psi _{\text{g}}$ in the growth configuration $\mathcal {B}_{g}$, where $\psi _0 = J^\text{g} \psi _\text{g}$. Based on our previous work^2^, we adopt a neo-Hookean strain energy function to describe the material behavior of brain tissue during cortical folding. This is formulated as follows,

3 $$\begin{aligned} \psi _{\text{g}}({\varvec{{F}}}^\text{e}) =&\frac{1}{2} \, \lambda \, \text {ln}^{2}(J^\text{e}) + \frac{1}{2} \, \mu \, \left[ \, {\varvec{{F}}}^\text{e}:{\varvec{{F}}}^\text{e} -3 -2 \text {ln}(J^\text{e}) \right] , \end{aligned}$$

where $\mu$ and $\lambda$ are the Lamé parameters that relate to the Poisson’s ratio $\nu$ in the linear regime as $\nu = \lambda / 2 [\lambda + \mu ]$.

**Fig. 2:**
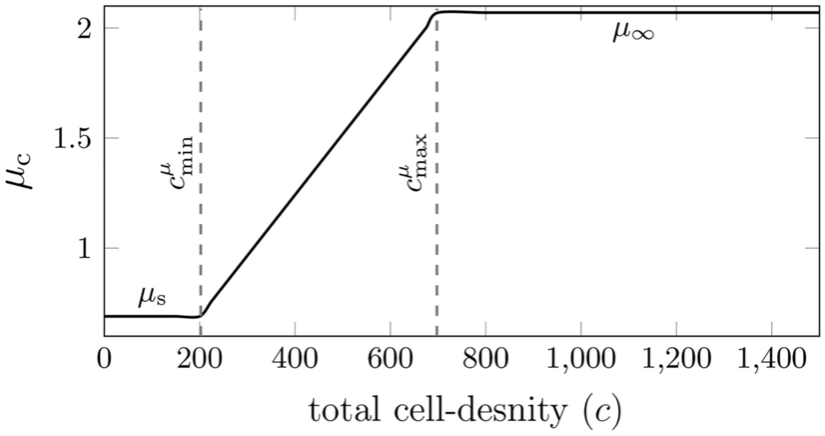
An idealized evolution of the cell-density- dependent cortical shear modulus $\mu _{\text{c}}$ during brain development, assumed to increase linearly from subcortical shear modulus $\mu _{\text{s}}$ to its final value corresponding to the fully developed cortex $\mu _{\infty }$.

According to our previous findings^35^, the human brain tissue tends to have a varying stiffness during development due to the changes in its local microstructure^36,37^. Consequently, we propose that the cortical shear modulus $\mu _{\text{c}}$ increases linearly from the subcortical shear modulus ($\mu _{\text{s}} = \text {constant}$) to that of a fully developed human cortex, $\mu _{\infty }$, such that,

4 $$\begin{aligned}&\displaystyle {\mu _{\text{c}}(c) = \text {min} \left( \, \mu _{\infty } \, , \,\mu _{s} + m^{\mu }_{\text{c}} \, \langle (c-c^{\mu }_{\text {min}} \,) \rangle \right) } \\&\text {with} \qquad \displaystyle {m^{\mu }_{\text{c}} = \frac{\mu _{\infty } - \mu _{\text{s}}}{c^{\mu }_{\text {max}} - c^{\mu }_{\text {min}}}}.\nonumber \end{aligned}$$

where $c^{\mu }_{\text {min}}$ is the minimum threshold, $c^{\mu }_{\text {max}}$ is the maximum threshold, and $\langle \rangle$ are Macaulay brackets that return its argument if the argument is positive and zero otherwise as shown in Fig. 2.

Adhering to the Clausius-Planck inequality, which presumes no internal energy dissipation within the hyperelastic materials^38^, we deduce the Cauchy stress as follows:

5 $$\begin{aligned} \varvec{\sigma }&= \frac{1}{J^\text{e}} \frac{\partial \psi _\text{g}({\varvec{{F}}}^\text{e})}{ \partial {\varvec{{F}}}^\text{e}} \cdot {{\varvec{{F}}}^\text{e}}^{T} = \frac{1}{J^\text{e}} \left[ \left[ \lambda \, \text {ln}(J^\text{e}) - \mu \right] {\varvec{{I}}} + \mu \, {\varvec{{b}}}^\text{e} \right] , \end{aligned}$$

where ${\varvec{{b}}}^\text{e} = {{\varvec{{F}}}^\text{e}} \cdot {{\varvec{{F}}}^\text{e}}^{T}$ is the elastic left Cauchy-Green tensor, and ${\varvec{{I}}}$ is the second order unit tensor.

#### Mechanical growth problem

The growth tensor ${\varvec{{F}}}^\text{g}$ is the key feature of the model, linking the mechanics problem with the cell-density problem. It thus plays a crucial role in controlling tissue deformation. We establish the mathematical formulation of the growth tensor based on the hypothesis of differential growth and guided by our understanding of cellular mechanisms^5,39,40^. During the early stages of human brain development, cellular processes, such as cell division, translocation of progenitor cells, and neuronal migration, all contribute to the subcortex’s isotropic expansion. As development continues, neurons settle in the cortex and start synapse formation, the development of neural connections results in more pronounced circumferential growth compared to radial growth^14,31^. This observation, which aligns with the differential growth hypothesis, is demonstrated in Fig. 1. Accordingly, we introduce independent growth multipliers $\vartheta ^\bot$ and $\vartheta ^\parallel$ in the circumferential and radial direction, respectively, as:

6 $$\begin{aligned} \vartheta ^\bot = \left[ 1+ \kappa ^\bot \, c \right] ^\alpha \quad \text {and} \quad \vartheta ^\parallel = \left[ 1+ \kappa ^\parallel \, c \right] ^\alpha , \end{aligned}$$

where the growth factors $\kappa ^\bot$, $\kappa ^\parallel$, and the growth exponent $\alpha$ determine the strength and shape of the coupling between the cell-density field and the mechanical field. Those multipliers are identical in the subcortex, denoted as $\vartheta ^\bot = \vartheta ^\parallel = \vartheta _{s}$, but differ in the cortex, where $\vartheta ^\bot> \vartheta _{s} > \vartheta ^\parallel$, as explained in more detail in Sect. “Model parameters”. Consequently, the growth tensor is written as,

7 $$\begin{aligned} {\varvec{{F}}}^\text{g} = \vartheta ^\bot \; \left[ {\varvec{{I}}} - {\varvec{{N}}} \otimes {\varvec{{N}}} \right] + \vartheta ^\parallel \; {\varvec{{N}}} \otimes {\varvec{{N}}}, \end{aligned}$$

where ${\varvec{{N}}}$ is the outward pointing normal vector in the reference configuration $\mathcal {B}_{0}$, linked to the spatial normal vector through ${\varvec{{N}}} = {\varvec{{F}}}^{-1} \cdot {\varvec{{n}}}$^12,35^.

#### Cell-density problem

To describe the behavior of each cell type involved in brain development, we additively decompose the cell-density field into four separate components. These components correspond to radial glial cells (RG), intermediate progenitor cells (IP), outer radial glial cells (ORG), and neurons (N), respectively, such that,

8 $$\begin{aligned} c = c_{\,\text {\tiny RG}} + c_{\,\text {\tiny IP}} + c_{\,\text {\tiny ORG}} + c_{\,\text {\tiny N}}. \end{aligned}$$

To cover the cell-density problem, we introduce a system of mass balance equations in the spatial configuration $\mathcal {B}_{t}$. Each equation is formulated to mimic the distinct behavior of a particular cell type. This system of equations is effectively summarized in a single mathematical expression as follows,

9 $$\begin{aligned} \frac{\dot{J}}{J} \, c_{ \, \bullet } + \dot{c}_{\, \bullet } = \text {div} \, {\varvec{{q}}}_{\, \bullet } ({\varvec{{x}}}, \, c_{\, \bullet }) + f_{\, \bullet } (t, \, c_{\, \circ }), \end{aligned}$$

where $\bullet$ serves as a placeholder for the specific cell type in question, ${\varvec{{q}}}_{\, \bullet }$ is the flux term, and $f_{\, \bullet }$ is the source term in the spatial configuration, with $\circ \in \{\text { RG, IP, ORG, N}\}$.

The flux term characterizes cell motion and is constructed as the sum of two distinct components: the advection term and the diffusion term, which are generally expressed as,

10 $$\begin{aligned} {\varvec{{q}}}_{\, \bullet }=&-\underbrace{c_{\, \bullet } \hat{{\textbf {v}}}_{\, \bullet }({\varvec{{x}}}, c_{\, \bullet })}_{\text {advection term}} +\underbrace{{\varvec{{d}}}^{\, \mathrm c}_{\, \bullet } ({\varvec{{x}}}, c_{\, \bullet }) \cdot \varvec{\nabla }_{{\varvec{{x}}}} \,c_{\, \bullet }}_{\text {diffusion term}}. \end{aligned}$$

In this formulation, the advection term describes cell transport, including translocation (such as for ORGCs), migration (as seen with neurons), or more regular movements (notably for IPCs). Meanwhile, the diffusion term describes cell dispersion, occurring either in the subcortex (for progenitor cells) or in the cortex (for neurons). The dynamics of transport and dispersion are cell-type specific and can be adjusted through certain parameters, which will be elaborated upon in Sect. “Model parameters”. In the advection term, the transport velocity vector $\hat{{\textbf {v}}}$ describes cell movement in radial direction, following the path of radial glial cell fibers towards the increasingly distant outer brain surface due to the growth^41^,

11 $$\begin{aligned} \hat{{\textbf {v}}}_{\, \bullet }({\varvec{{x}}}, c_{\, \bullet }) = \mathcal {H}(c_{\, \bullet } - c_{0}; \gamma ^\text{c} ) \, v_{\, \bullet }({\varvec{{x}}}) \, \frac{{\varvec{{n}}}}{\Vert {\varvec{{n}}} \Vert }, \end{aligned}$$

where $v_{\, \bullet }$ defines the speed of transport, and the current norm vector ${\varvec{{n}}}$ determines the direction of transport. The latter depends on the mechanical deformation through the push forward operator, i.e., ${\varvec{{n}}} = {\varvec{{F}}} \cdot {\varvec{{N}}}$. Here, the term $1/{\Vert {\varvec{{n}}} \Vert }$ serves to normalize the direction vector. Finally, we use the nonlinear regularized Heaviside function $\mathcal {H}(c_{\, \bullet } - c_{0}; \gamma ^c )$, with the threshold $c_{0}$ and exponent $\gamma ^c$, to model the impact of adaptive transport based on type-specific cell density. It ensures a smooth increase in the amount of transport as the cell density rises. The general formulation of this function is given by,

12 $$\begin{aligned} \mathcal {H}(\circ ; \gamma ) =\frac{e^{\gamma \, \circ }}{ 1 + e^{\gamma \, \circ }}. \end{aligned}$$

Assuming isotropic diffusion, the diffusion tensor may be written as,

13 $$\begin{aligned} {\varvec{{d}}}^{\, \mathrm c}_{\, \bullet }({\varvec{{x}}}, c_{\, \bullet }) = \left[ {d}^{\, \mathrm c}_{\, \bullet } ({\varvec{{x}}})+ \nu _{\, \bullet }^{\, \mathrm c}(c_{\, \bullet }) \right] \, {\varvec{{I}}} \end{aligned}$$

where ${d}^{\, c}_{\, \bullet }$ is the type-specific diffusivity that is a function of the spatial position, and $\nu _{\, \bullet }^{\, c}$ is an artificial (numerical) diffusivity. Although this artificial diffusivity has no physical meaning, it is intended to ensure numerical stability within the advection-diffusion equation, addressing the disparity between the advection and diffusion terms in the transition area between the cortex and subcortex at the onset of transition. To compute this value, we follow the streamline upwind Petrov-Galerkin (SUPG) method. For more computational details, we refer to^42^.

Based on the lineage relationships between the different cell types under consideration, we define the source term in Eq. (9) as follows,

14 $$\begin{aligned} f_{\, \bullet } (t, \, c_{\, \circ }) = \sum _{\begin{array}{c} \circ \in \{ \text {\tiny RG, IP, ORG, N} \} \end{array}} G_{ \, \bullet } (\circ , \text {P}(t)) \, c_{\, \circ }, \end{aligned}$$

where the summation $\sum$ extends over the four cell types, $G_{ \, \bullet } (\circ , \text {P})$ is a division ratio between the cell types $\bullet$ and $\circ$ in the particular division phase $\text {P}$, and $c_{\, \circ }$ denotes the density of cell type $\circ$. More details regarding the source term will be given in the model parameter Sect. “Model parameters”.

#### Discretization in time

Since the system of cell density balance equations given in 9 includes two time-dependent components, namely the Jacobian and the type-specific cell density, time discretization is essential. In our approach, we have, in addition to the cell density field, the nonlinear deformation field that requires a nonlinear solver. Therefore, we can employ the nonlinear solver to implicitly calculate the time-dependent terms using an Euler discretization scheme^13^. We subdivide the time domain $\mathcal {T}$ into $\mathrm n_{\, \mathrm st}$ intervals such that,

15 $$\begin{aligned} \mathcal {T} = \bigcup _{\mathrm{n =1}}^{{\text{n}}_{\, \text{st}}} \left[ t_\mathrm{n-1} , t_{\text{n}}\right] , \end{aligned}$$

and obtain,

16 $$\begin{aligned} \dot{c}_{ \, \bullet }= \displaystyle {\frac{c_{{\, \bullet }, \, \text{n}}- c_{{\, \bullet }, \, \mathrm{n-1}}}{\Delta t}}, \qquad \text {and} \qquad \dot{J} = \displaystyle {\frac{J_{\text{n}}-J_{\mathrm{n-1}}}{\Delta t}}, \end{aligned}$$

where $c_{{\, \bullet }, \, \text{n}}$ and $J_{\text{n}}$ are the unknown values at the current time step, $c_{{\, \bullet }, \, \mathrm{n-1}}$ and $J_{\mathrm{n-1}}$ are the known values from the previous time step, and $\Delta t = t_{\text{n}}-t_{\mathrm{n-1}}$ is the time increment. As a result, we derive the fully time-discretized system of equations for the cell density balance as follows,

17 $$\begin{aligned}&\displaystyle {\frac{J_{\text{n}}-J_{\mathrm{n-1}}}{J \, \Delta t}} \, c_{ \, \bullet , \, \mathrm n} + \displaystyle {\frac{c_{{\, \bullet }, \, \text{n}}- c_{{\, \bullet }, \, \mathrm{n-1}}}{\Delta t}} = \text {div} \, {\varvec{{q}}}_{\, \bullet }({\varvec{{x}}}, \, c_{{\, \bullet }, \, \text{n}}) + f_{ \, \bullet }(t_{\text{n}}, \, c_{{\, \bullet }, \, \mathrm{n-1}}). \end{aligned}$$

The source term is approximated explicitly as,

18 $$\begin{aligned}&f_{\, \bullet } (t_{\text{n}}, \,c_{{\, \circ }, \, \mathrm{n-1}}) = \sum _{\begin{array}{c} \circ \in \{ \text {\tiny RG, IP, ORG, N} \} \end{array}} \Delta t \, G_{ \, \bullet } (\circ , \text {P}(t_{\text{n}})) \, c_{\, \circ , \, \mathrm{n-1}}. \end{aligned}$$

#### Geometry and discretization in space

**Fig. 3:**
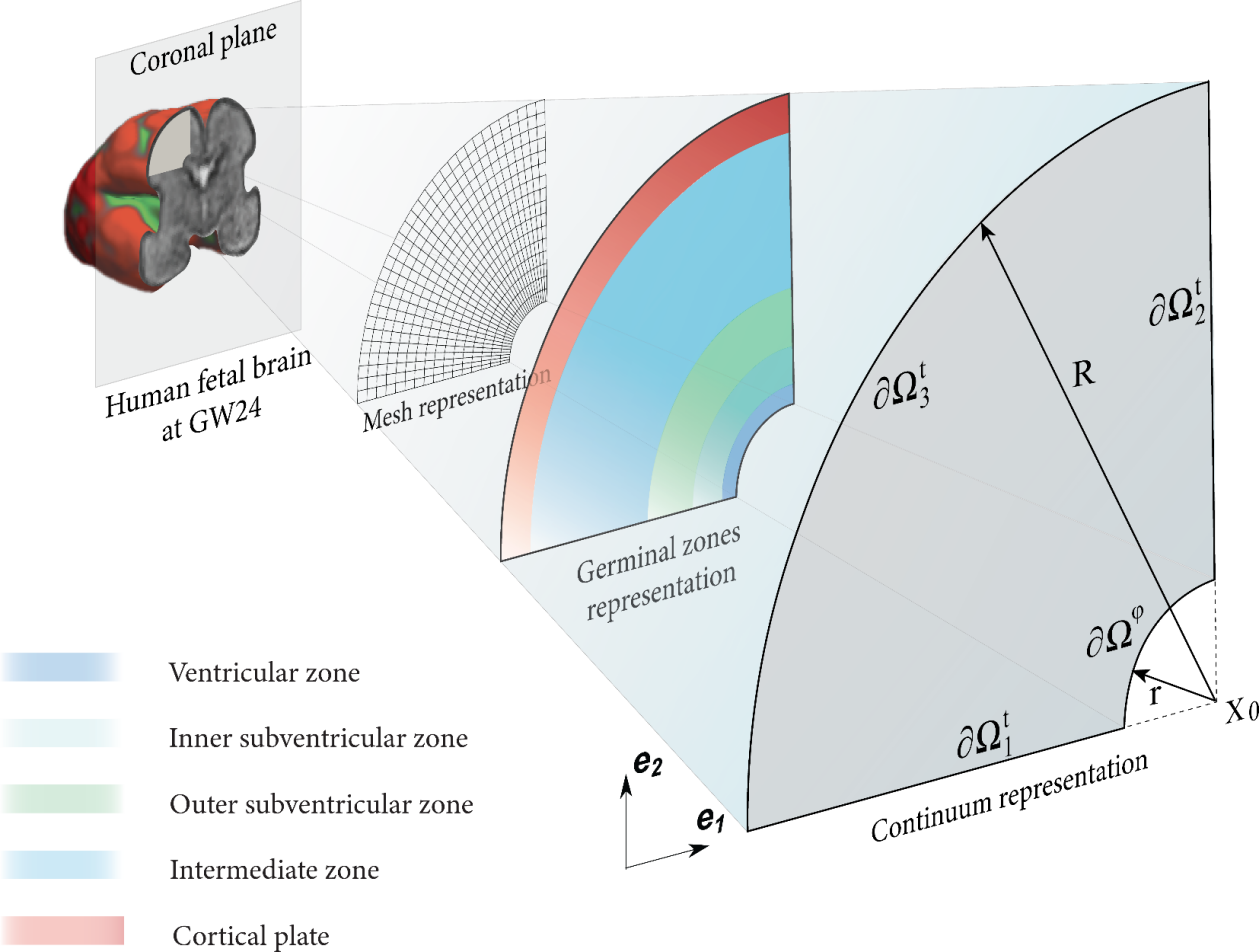
The simulation domain represents a 2D exemplary part of a coronal slice at gestational week 24 with the outer radius *R* and ventricular radius *r*. The schematic provides various views of the domain through representations. The continuum representation demonstrates the Dirichlet $\partial \varvec{\Omega }^\varphi$ and Neumann boundaries $\partial \varvec{\Omega }^\text{t}$. The germinal zones representation highlights the spatial locations of these zones within the simulation domain. The mesh representation illustrates the initial mesh of the domain.

To systematically study the interaction between the proliferation of different cell types in the developing human brain and cortical folding, we chose to use a two-dimensional domain that represents an exemplary part of a coronal slice at gestational week 24, as shown in Fig. 3. The selected domain facilitates comparison with magnetic resonance (MR) images and histologically stained sections of fetal brains (HBS)^35^, while simultaneously lowering computational cost, making it an optimal choice for our application. The following Dirichlet and Neumann boundary conditions are applied on the domain,

19 $$\begin{aligned}&\varvec{\varphi }({\varvec{{x}}}) = {\varvec{{0}}}&\text {for} \hspace{25pt} {\varvec{{x}}}\in \partial \varvec{\Omega }^{\varphi } \nonumber \\&\varphi _2 ({\varvec{{x}}})= 0&\text {for} \hspace{25pt} {\varvec{{x}}}\in \partial \varvec{\Omega }^\text{t}_1 \nonumber \\&\varphi _1 ({\varvec{{x}}})= 0&\text {for} \hspace{25pt} {\varvec{{x}}}\in \partial \varvec{\Omega }^\text{t}_2 \nonumber \\&\varvec{\sigma }\cdot {\varvec{{n}}}={\varvec{{0}}}&\text {for} \hspace{25pt} {\varvec{{x}}}\in \partial \varvec{\Omega }^\text{t}_3 \nonumber \\&{\varvec{{q}}}_{\, \bullet }\cdot {\varvec{{n}}}={\varvec{{0}}}&\text {for} \hspace{25pt} {\varvec{{x}}}\in \partial \varvec{\Omega }^\text{t}_3 \end{aligned}$$

where $\partial \varvec{\Omega }^\varphi$ is the Dirichlet boundary and $\partial \varvec{\Omega }^\text{t}$ are the Neumann boundaries satisfying $\partial \varvec{\Omega }^{ \mathrm t} \cup \partial \varvec{\Omega }^\varphi = \partial \varvec{\Omega }$ and $\partial \varvec{\Omega }^\text{t}\cap \partial \varvec{\Omega }^\varphi = \emptyset$. Here, $\partial \varvec{\Omega }^\text{t}_1 = \{ {\varvec{{x}}} \in \partial \varvec{\Omega }^\text{t} \, \big | \, x_2 = 0 \}$ and $\partial \varvec{\Omega }^\text{t}_2 = \{ {\varvec{{x}}} \in \partial \varvec{\Omega }^\text{t} \, \big | \, x_1 = 0 \}$, where $\varphi _1$ and $\varphi _2$ are the first and second component of the deformation map. In addition, we segment the simulation domain into five neighboring sections, representing the germinal zones within the brain (see Fig. 3). Mathematically, each zone is distinguished by a specific radius; $r_{\text {vz}}$ represents the upper limit of the ventricular zone, $r_{\text {isvz}}$ represents the upper boundary of the inner subventricular zone, $r_{\text {osvz}}$ represents the upper and final boundary of the outer subventricular zone, while $r_{\text {cp}}$ represents the lower boundary of the cortex. Finally, we discretize the geometry into a regular finite element mesh and linearize the nonlinear coupled problem using the Newton-Raphson method. Accordingly, we end up with the following global system of algebraic equations that can be solved numerically,

20 $$\begin{aligned} \underbrace{\begin{bmatrix} \textbf{K}^{\varphi \, \varphi } & \textbf{K}^{\varphi \, \text {\tiny RG}} & \textbf{K}^{\varphi \, \text {\tiny IP}} & \textbf{K}^{\varphi \, \text {\tiny ORG}} & \textbf{K}^{\varphi \, \text {\tiny N}}\\ \textbf{K}^{ \text {\tiny RG} \, \varphi }& \textbf{K}^{\text {\tiny RG} \, \text {\tiny RG}} & 0 & 0& 0\\ \textbf{K}^{ \text {\tiny IP} \, \varphi } & 0& \textbf{K}^{{\text {\tiny IP}} \,{\text {\tiny IP}}} & 0& 0\\ \textbf{K}^{ {\,\text {\tiny ORG}}\, \varphi } & 0 & 0& \textbf{K}^{{\text {\tiny ORG}}{\,\text {\tiny ORG}}} & 0\\ \textbf{K}^{ {\text {\tiny N}} \, \varphi } & 0 & 0& 0& \textbf{K}^{{\text {\tiny N}}\,{\text {\tiny N}}} \\ \end{bmatrix}}_{\textbf{K}} \cdot \underbrace{\begin{bmatrix} \Delta \, \varvec{\varphi }\\ \Delta \, c_{\,\text {\tiny RG}} \\ \Delta \, c_{\,\text {\tiny IP}} \\ \Delta \, c_{\,\text {\tiny ORG}} \\ \Delta \, c_{\,\text {\tiny N}} \end{bmatrix}}_{\Delta \, \varvec{\phi }} = \underbrace{\begin{bmatrix} \textbf{R}^{\varphi }\\ \text {R}^{c_{\,\text {\tiny RG}}}\\ \text {R}^{c_{\,\text {\tiny IP}}}\\ \text {R}^{c_{\,\text {\tiny ORG}}}\\ \text {R}^{c_{\,\text {\tiny N}}} \end{bmatrix}}_{\textbf{R}}, \end{aligned}$$

where $\textbf{K}$, $\Delta \, \varvec{\phi }$, and $\textbf{R}$ are the total tangent matrix, total incremental solution vector, and right-hand side residual vector, respectively. Importantly, the zeros in the tangent matrix indicate the absence of direct coupling (implicit) between the different cell density components. Rather, the coupling is addressed indirectly (explicitly) via the source terms, which appear on the right-hand side in the residual vector.

### Model parameters

The developmental dynamics of the human brain and its heterogeneous microstructure lead to a non-uniform distribution of model parameters throughout the simulation domain, i.e., the shear modulus, growth factors, amount of transport, and diffusivity. These parameters vary significantly across the different brain zones^2^, introduced in Fig. 1. Furthermore, the real human brain does not show distinct boundaries between its zones. To address this issue, on the one hand, and ensure numerical stability, on the other hand, we use the Heaviside function, introduced in equation (12) to model a smooth transition at the interfaces between zones. Here, we define the Heaviside function as a function of the radius $r_{i}$ of a material point ${\varvec{{X}}}$ relative to the geometric center ${\varvec{{X}}}_0$, calculated as $r_{i} = \parallel {\varvec{{X}}} - {\varvec{{X}}}_0 \parallel _{2}$.

**Fig. 4:**
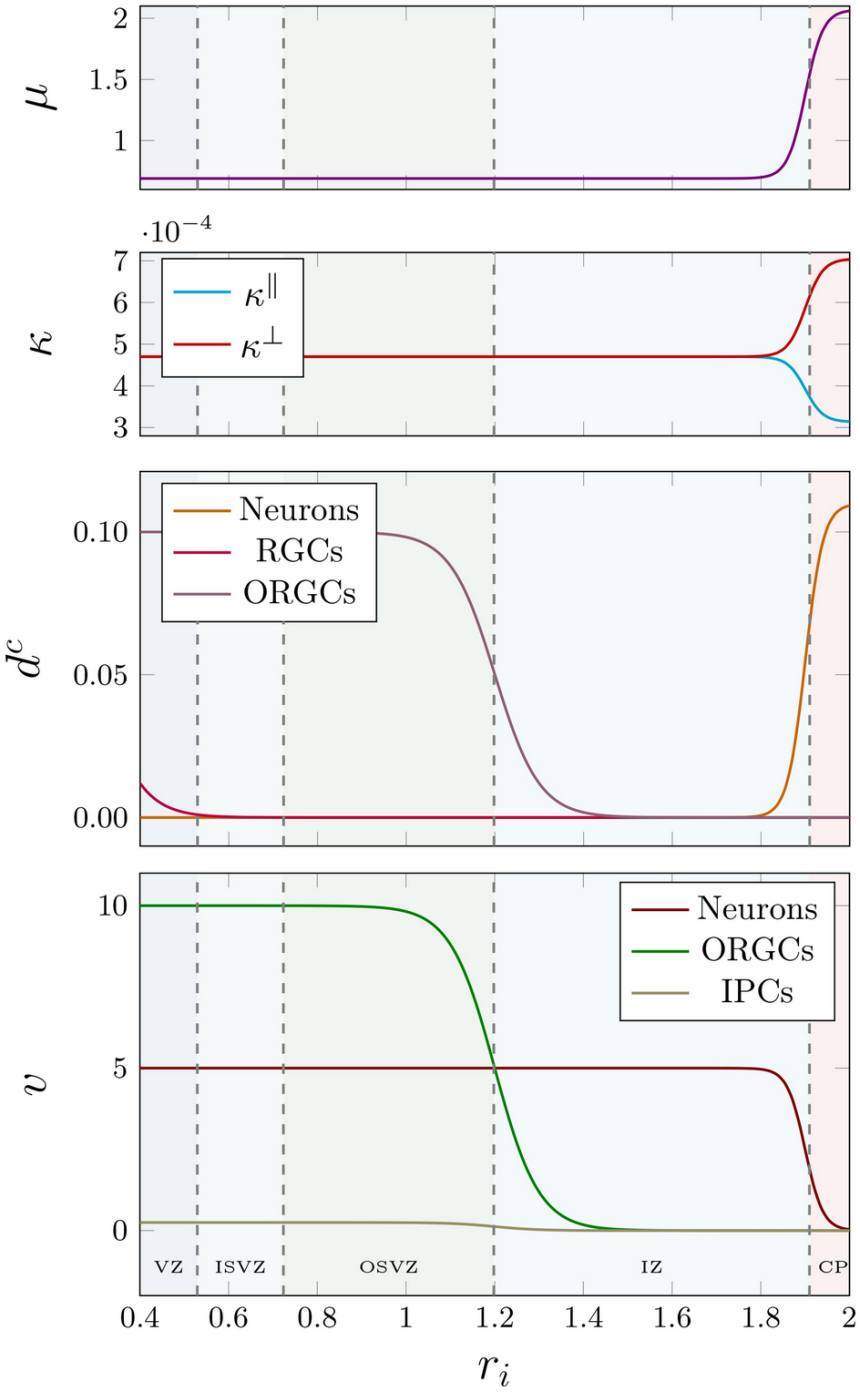
Distribution of model parameters along the domain’s radial direction from the ventricular zone to the outer cortical surface: shear modulus $\mu$; tangential and radial growth factors $\kappa ^\bot \, ,\, \kappa ^\parallel$; diffusivity $d^\text{c}$ of neurons, radial glial cells (RGCs), and outer radial glial cells (ORGCs), intermediate progenitor cells (IPCs); migration speed *v* of neurons and translocation speed of outer radial glial cells and intermediate progenitor cells. The corresponding model parameters are summarized in Table 2.

Given that the cortex is $\beta _{\mu }$ times stiffer than the subcortex, we define the transition of the shear modulus at the cortex-subcortex boundary $r_{\text {cp}}$, as,

21 $$\begin{aligned} \mu (r_{\text{i}}) = \mu _{\text{s}}+\left[ \mu _{\text{s}} \left[ \beta _{\mu}-1 \right] \times \mathcal {H} (r_{\text{i}} - r_{\text {cp}} ; \gamma ^\text{r}_\text {cp})\right] , \end{aligned}$$

where $\beta _{\mu } = \mu _{\infty } / \mu _{\text{s}} \ge 1$ denotes the stiffness ratio between cortex and subcortex, and $\gamma ^\text{r}_{\text {cp}}$ represents the Heaviside spatial exponent at the cortex. The distribution of the shear modulus along the radial direction of the geometry is illustrated in Fig. 4 (top). In the same manner, to satisfy the differential growth discussed in Sect. “Mechanical growth problem”, the growth factors in the circumferential and radial directions $\kappa ^\bot$ and $\kappa ^\parallel$, respectively, are modeled as,

22 $$\begin{aligned}&\kappa ^\bot (r_{\text{i}}) = \kappa _{\text{s}}+\left[ \kappa _{\text{s}} \left[ \beta _{\kappa }-1 \right] \mathcal {H} (r_{i} - r_{\text {cp}};\gamma ^\text{r}_\text {cp} ) \right] \quad \text {and} \nonumber \\&\kappa ^\parallel (r_{\text{i}}) = \kappa _{\text{s}}+\left[ \kappa _\text{s} \left[ \frac{1}{\beta _{\kappa }}-1 \right] \mathcal {H} (r_{\text{i}} -r_{\text {cp}};\gamma ^\text{r}_\text {cp} ) \right] , \end{aligned}$$

with the growth ratio $\beta _{\kappa }$ given as,

23 $$\begin{aligned} \beta _{\kappa }= \frac{ \kappa ^\bot }{ \kappa _{\text{s}}} = \frac{ \kappa _{\text{s}}}{\kappa ^\parallel } \ge 1. \end{aligned}$$

Again, Fig. 4 shows how the growth factor differentiates in the cortex into circumferential and radial factors. Finally, we introduce the parameters for the advection-diffusion equation in Sect. “Cell-density problem” to capture the distinct behaviors of each cell type. IPCs are characterized by a notably slow movement to occupy the space between the ventricular zone and the outer subventricular zone,

24 $$\begin{aligned} v_{\, \text {\tiny IP}}(r_{\text{i}}) = v_{\, \text {\tiny IP}} \left[ 1- \mathcal {H} (r_{\text{i}} - r^\text{t}_{\text {osvz}}; \gamma ^\text{r}) \right] , \end{aligned}$$

while ORGCs are translocated directly after they are born to the outer subventricular zone,

25 $$\begin{aligned} v_{\, \text {\tiny ORG}}(r_{\text{i}}) = v_{\, \text {\tiny ORG}} \left[ 1- \mathcal {H} (r_{\text{i}} - r^\text{t}_{\text {osvz}}; \gamma ^\text{r}) \right] . \end{aligned}$$

On the other hand, neurons migrate towards the cortex,

26 $$\begin{aligned} v_{\, \text {\tiny N}}(r_{\text{i}}) = v_{\, \text {\tiny N}} \left[ 1- \mathcal {H} (r_{\text{i}} - r_{\text {cp}}; \gamma _{\text {cp}}^\text{r}) \right] . \end{aligned}$$

The second parameter that needs to be adjusted is the diffusivity. RGCs undergo diffusion after division within the ventricular zone,

27 $$\begin{aligned} d_{\, \text {\tiny RG}}^{\, \mathrm c}(r_{\text{i}})&= d^{\, \mathrm c} \, \left[ 1- \mathcal {H} (r_{\text{i}} - r_{\text {vz}}; \gamma ^\text{r}) \right] . \end{aligned}$$

Similarly, IPCs and ORGCs disperse within their respective effective zones, namely between the ventricular zone and the outer subventricular zone,

28 $$\begin{aligned} d_{\, \text {\tiny ORG}}^{\, \mathrm c}(r_{\text{i}}) = d_{\, \text {\tiny IP}}^{\,\mathrm c}(r_{\text{i}})&= d^{\, \mathrm c} \, \left[ 1- \mathcal {H} (r_{\text{i}} - r^\text{t}_{\text {osvz}}; \gamma ^\text{r}) \right] . \end{aligned}$$

In contrast, neurons exhibit a pure migration behavior within the subcortex. Once they reach the cortex, they diffuse to occupy the cortical layer,

29 $$\begin{aligned} d_{\, \text {\tiny N}}^{\, \mathrm c}(r_{\text{i}})&= d_{\, \text {\tiny N}}^{\, \mathrm c} \, \mathcal {H} (r_{\text{i}} - r_{\text {cp}};\gamma _{\text {cp}}^\text{r} ). \end{aligned}$$

The radial distributions of the type-specific amount of transport and diffusivity are demonstrated in Fig. 4 (bottom).

To account for the impact of the mitotic small translocation behavior of ORGCs, which significantly pushes the outer boundary of the subventricular zone between gestational weeks 11 and 24^23^, we define the time-dependent radius $r^\text{t}_{\text {osvz}}$ in Eqs. 24 , 25, and 26. This radius is formulated as a linear function of time within the specified period, increasing from the inner subventricular zone upper boundary until reaching its maximum value, such that

30 $$\begin{aligned} r^\text{t}_{\text {osvz}} = \text {min} \{ r_{\text {osvz}} \, , \, r_{\text {isvz}} + m_{\text {mst}} \, \langle t - t_{\text {P2}}\rangle \}, \end{aligned}$$

where $r_{\text {osvz}}$ is the upper and final boundary of the outer subventricular zone, as mentioned in Sect. “Geometry and discretization in space”, $m_{\text {mst}}$ is the mitotic small translocation constant and $t_{\text {P2}}$ is the end time of the second phase. The constant $m_{\text {mst}}$ is determined based on the observed distance between the inner and outer subventricular zones in histologically stained sections of the human fetal brain from gestational weeks 17–32. This constant is calculated as the ratio of this observed distance over the corresponding time period.

**Fig. 5:**
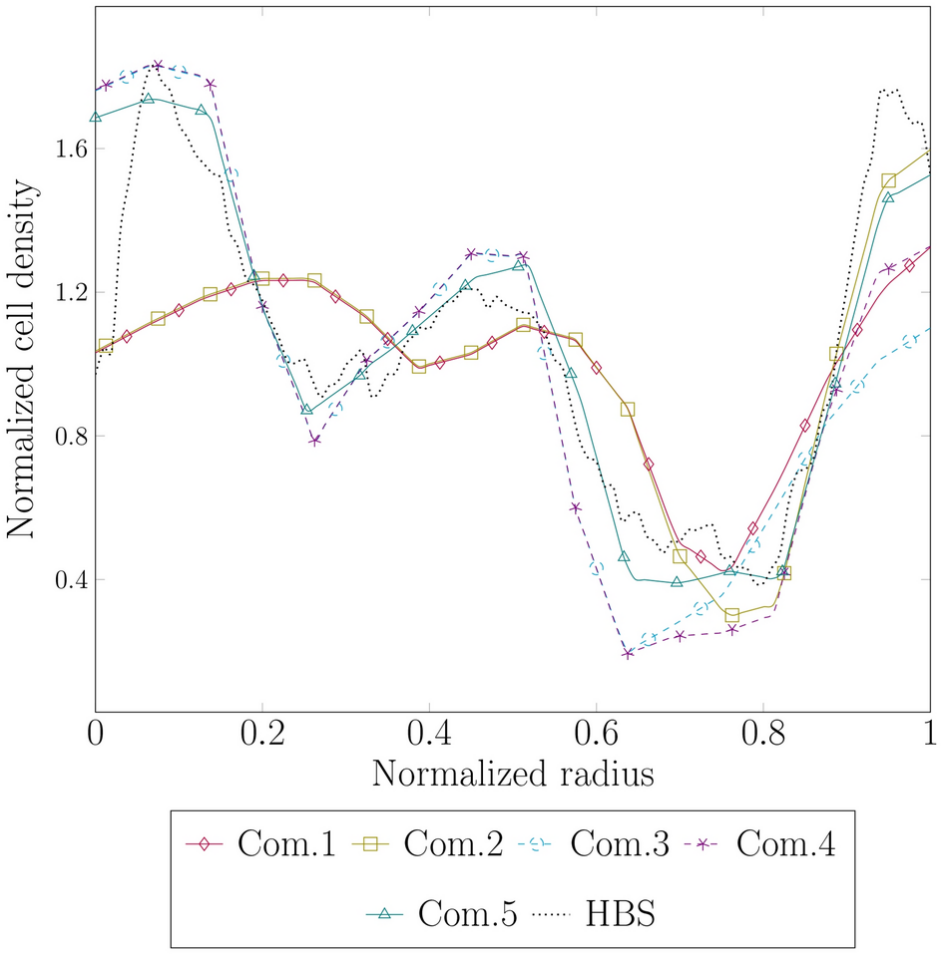
Evolution of the cell density distribution along the normalized radius from the ventricular surface to the outer brain surface for numerical simulation results corresponding to gestational week 18 across different radial distributions of parameters, as introduced in Table 1, alongside a stained human brain section at gestational week 18 (HBS). The cell density is normalized to its average value. Combinations 1 to 5 display the simulation results, each employing a distinct set of exponents in the parameters’ radial distribution equations. The corresponding model parameters are summarized in Table 2.

In the equations defining the radial distribution of all previous parameters, $\gamma ^\text{r}$ serves as the general spatial exponent for the Heaviside function, while $\gamma _{\text {cp}}^\text{r}$ specifically applies at the subcortex-cortex interface. Both exponents are critical for controlling how smoothly parameters change at the boundaries between different zones. To calibrate these exponents, we analyze how well our simulation results match with histologically stained images of the human fetal brain around gestational week 18. Regarding the method used in preparing, staining, and processing these sections, please see^14^. Figure 5 illustrates a comparison between the observed cell density distribution along a trajectory from the ventricular surface to the outer brain surface for the stained section and simulation results for five different exponent combinations, as listed in Table 1. To enhance comparability and avoid differences in the dimensions between the histologically stained sections and the simulation domain, we normalize the domain’s radius according to the extension from the ventricular to the outer cortical surface in the stained sections. Furthermore, cell density is normalized relative to its average value. The distribution characterized by $\gamma ^\text{r}=20$ and $\gamma _{\text {cp}}^\text{r} = 50$ closely replicates the trend observed in the histologically stained sections. The distinct peaks at the ventricular zone, outer subventricular zone, and cortex align precisely with those identified in the stained images. Similarly, the simulation results for the parameter combination 5 and the corresponding distribution accurately depict the observed local minima at the inner subventricular zone and intermediate zone.

**Table 1:** Exponents of the parameters’ radial distribution equations corresponding to the distributions shown in Fig. 5.

Combination	$\gamma ^{ \mathrm r}$	$\gamma ^\text{r}_{\text {cp}}$
Combination 1	10	10
Combination 2	10	30
Combination 3	20	10
Combination 4	20	30
Combination 5	20	50

All model parameters are summarized in Table 2. In general, the mechanical parameters are adopted from our prior studies on human brain tissue^2,9,34^. Geometric parameters, in contrast, are evaluated from histologically stained sections of the human fetal brain, which reveal the boundaries of the germinal layers. However, an initial radius estimate is made to ensure optimal visualization of the results. In the results Sect. “Results and discussion”, we calibrate our simulation results for convenient comparison with Magnetic resonance images. Growth parameters were originally obtained from^12^, yet they have been adapted to guarantee algorithmic convergence. Finally, the parameters for the cell density problem have been carefully adjusted experimentally to better align with actual human brain development, while preserving numerical stability.

**Table 2:** Model parameters.

Geometry parameters				Mechanical problem parameters			
Parameter		Value	Unit	Parameter		Value	Unit
Outer brain radius	*R*	2	mm	Cortex shear modulus	$\mu _{\infty }$	2.07	kPa
inner brain radius	*r*	0.4	mm	Poisson ratio	$\nu$	0.38	–
VZ radius	$r_{\text {vz}}$	0.5	mm	stiffness ratio	$\beta _{\mu }$	3	–
ISVZ radius	$r_{\text {isvz}}$	0.8	mm	maximum threshold	$c^{\mu }_{\text {max}}$	700	mm^−2^
Maximum OSVZ radius	$r_{\text {osvz}}$	1.2	mm	minimum threshold	$c^{\mu }_{\text {min}}$	200	mm^−2^
Cortex radius	$r_{\text {cp}}$	1.9	mm				
MST factor	$m_{\text {mst}}$	0.02	mm d^−1^				

Cell density problem parameters				Mechanical growth problem parameters			
Parameter		Value	Unit	Parameter		Value	Unit
Neurons migration speed	$v_{\,\text {\tiny N}}$	5	mm d^−1^	Growth parameter	$\kappa _{\text{s}}$	$4.07e^{-4}$	mm^2^
ORG translocation speed	$v_{\, \text {\tiny ORG}}$	10	mm d^−1^	Growth exponent	$\alpha$	1.65	–
IP translocation speed	$v_{\, \text {\tiny IP}}$	0.025	mm d^−1^	Growth ratio	$\beta _{\kappa }$	1.5	–
Heaviside threshold	$c_0$	500	mm^−2^				
Heaviside exponent	$\gamma ^\text{c}$	0.008	–				
Neurons diffusivity	$d_{\, \text {\tiny N}}^{\, \mathrm c}$	0.11	mm^2^ d^−1^				
other cell-types diffusivity	$d^{\, \mathrm c}$	0.1	mm^2^ d^−1^				
Stabilization constant	$\beta$	0.1	–				

**Fig. 6:**
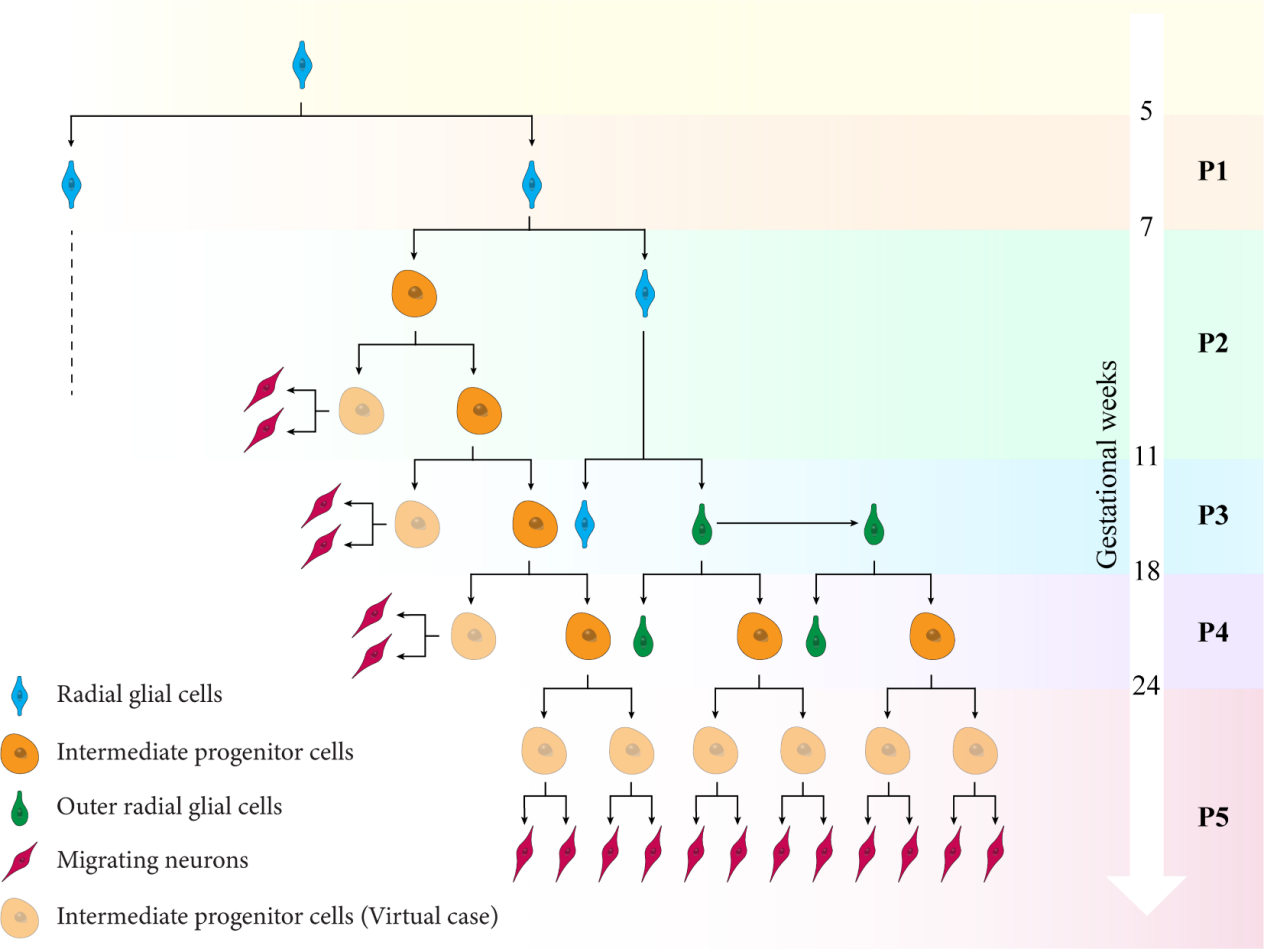
The lineage tree and progression among the four fundamental neural progenitor cells during various gestational weeks in human brain development. This lineage tree is organized into five primary phases, designated P1 through P5, advancing from the early to the later stages of development. The term “virtual case of the intermediate progenitor cells” describes a scenario that physically transpires within the brain during development, yet is not accounted for in numerical simulations.

To complete the set of model parameters, we need to define the division ratio between cell types for each division phase, i.e., $G_{ \, \cdot } (\circ , \text {P})$. This step required an initial understanding of the lineage relationships between different cell types. Figure 6 illustrates the lineage tree and progression among the four considered cell types during the various gestational weeks. According to the literature^3,5,24,43–46^, the lineage tree can be organized into five primary phases, designated from P1 through P5, advancing from the early to the later stages of development. Additionally, defining a timeline for our simulation is essential; for this purpose, we establish a formula linking gestational weeks $t_{\text {GW}}$ to computational time *t*, expressed as

31 $$\begin{aligned} t_{\text {GW}} = 0.3 \times t + 4. \end{aligned}$$

The division ratios between cells across different division phases are detailed in Supplementary Table 1. Here, we provide an example of how we determined the division ratios. Considering the division of RGCs during the third phase (P3), each RGC divides into one RGC and one ORGC, represented as $G_{\text {\tiny RG}} (\text { RG}, \text { P3}) = 1$ and $G_{\text {\tiny ORG}} (\text { RG}, \text { P3}) = 1$, respectively. ORGCs then divide again, each giving rise to another ORGC, thus $G_{\text {\tiny ORG}} (\text { ORG}, \text { P3}) = 1$. Meanwhile, each IPC divides to form two new IPCs, and one of these IPCs further divides to produce two neurons, leading to $G_{\text {\tiny IP}} (\text { IP}, \text { P3}) = 1$ and $G_{\text {\tiny N}} (\text { IP}, \text { P3}) = 2$. Following the calculation of division ratios, the source term can be computed using Eq. (14), as outlined in the Supplementary Material.

## Results and discussion

In a first step, we compare our simulation results with the folding pattern observed on magnetic resonance imaging (MRI) data obtained from neonates, including those born prematurely between postconceptual weeks 23 to 44. The imaging data is provided by the developing Human Connectome Project (dHCP), King’s College London - Imperial College London - Oxford Consortium^47^. The MRI acquisition was conducted on a 3T Philips scanner using a dedicated brain imaging system^48^. The fetuses were imaged in utero and the neonates were imaged in natural sleep. The images were reconstructed with motion correction to alleviate the effects of head motion of the participants during image acquisition^49^. In this work, we use T2-weighted (T2w) brain MRI and corresponding cortical surfaces derived from the dHCP data release^47^, which has passed the manual quality control performed by domain experts^47^. For detailed information regarding data acquisition methods and pre-processing of the datasets, we refer to^50–53^.

In a second step, we discuss the spatial and temporal distributions of the type-specific density and demonstrate how our computational model can be used for cellular deconvolution of the histologically stained images of human fetal brain sections.

Finally, we investigate the folding patterns under different parameter combinations of the growth and stiffness ratios, considering two distinct cases regarding cortical stiffness: a variable stiffness case, as introduced in Sect. “Mechanics problem”, and a constant stiffness case, where $\mu _{\text{c}} = \mu _{\infty } = \text {const.}$. Despite our prior emphasis on the tendency of the human brain to exhibit varying stiffness during development, we include both cases here to validate our hypothesis, now by taking into account deeper biological insights^35,54^. It is worth noting that all simulation results presented in this section are obtained using the parameters introduced in Table 2, unless explicitly specified otherwise.

### Comparative visualization: MRI sections vs. simulation results

**Fig. 7:**
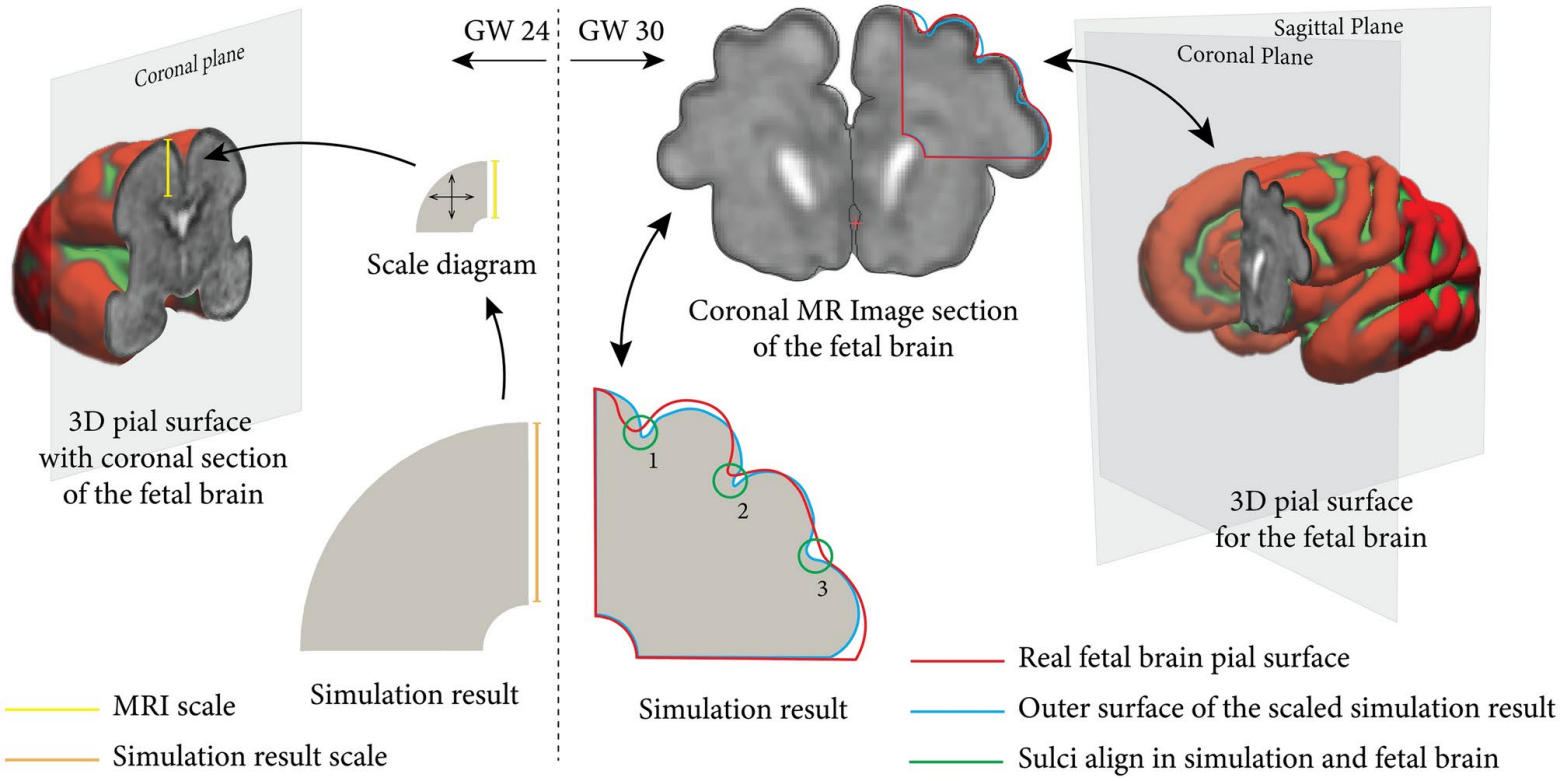
Comparability of magnetic resonance (MR) images and simulations. Left: The scaling procedure of the simulation result at gestational week (GW) 24 to match the coronal section of the real human brain on MR images. The diagram in the center illustrates the isotropic scaling of the simulation result concerning the distance between the ventricular surface and the pial surface (the outer surface between brain tissue and the surrounding cerebrospinal fluid) of MR images. Right: Comparative visualization of fetal brain development at gestational week 30 between magnetic resonance imaging (MRI) section and scaled simulation results. The left panel shows a coronal MR image section of the fetal brain, with key areas indicated. The lower illustration demonstrates the simulation results with overlaying outlines-red for the actual pial surface of the fetal brain and blue for the outer surface from the simulation results. The right panel presents a 3D representation of the fetal brain’s pial surface, indicating the correlation between the simulation and the full 3D anatomical structure. The simulation result corresponds to a stiffness ratio of 3 and a growth ratio of 1. The following software was used to generate this figure: Freeview software (V3.0)^61^, Adobe Illustrator (V28.7.1), ParaView (V5.11), and ImageJ software (V1.53)^60^.

In order to ensure comparability between the simulation results and MRI images, it is necessary to establish a consistent scaling. Therefore, we align the scale of the simulation result for gestational week 24 (obtained according to the model timeline specified in Eq. 31) with the distance between the ventricular surface and the pial surface of MR images for the same week, as illustrated in Fig. 7, left. We then track the changing geometry and increasing radii in subsequent gestational weeks.

Figure 7, right, illustrates a comparative visualization of fetal brain development at gestational week 30, showcasing both the MRI section and the corresponding scaled simulation results. The simulation output, corresponding to a stiffness ratio of three and growth ratio of one, match closely with the folding pattern observed in the coronal section of the human brain on MR images. In the actual human brain section, three grooves (sulci) are evident, denoted as 1 to 3 in Figure. Notably, sulci 1 and 2 exhibit greater depths compared to sulcus 3, suggesting the occurrence of a secondary mechanical instability during development^55,56^. The simulation accurately captures the positions of these sulci, indicating its ability to provide insights into the biological processes contributing to this mechanical instability, as further elucidated in subsequent sections.

**Fig. 8:**
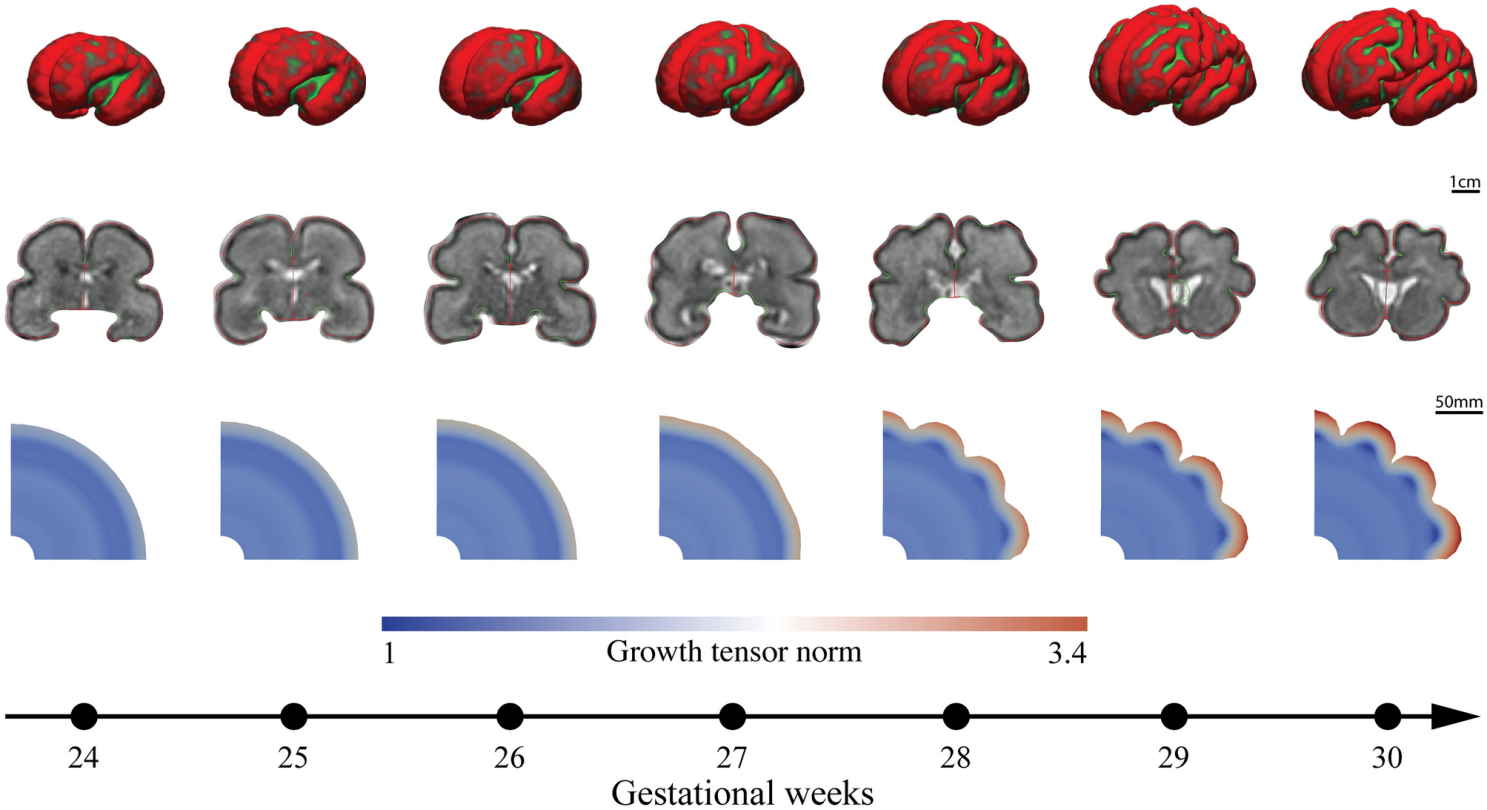
Exemplary progression of cortical folding from gestational week 24–30 on a part of the coronal section of the developing human brain. The upper row depicts the fetal brain’s surface development throughout this timeframe, with red areas indicating concave outward regions and green areas representing concave inward regions reconstructed from magnetic resonance imaging (MRI) data. The central row displays the respective coronal MRI sections of the fetal brain. The bottom row illustrates the simulation results corresponding to a stiffness ratio of 3 and a growth ratio of 1. The color gradients in the simulation results reflect the Frobenius norm of the growth tensor. The result captures the developmental trajectory well and resembles cortical folding patterns observed in the MRI sections of the human brain. The following software was used to generate this figure: Freeview software (V3.0)^61^, Adobe Illustrator (V28.7.1), and ParaView (V5.11).

To further elucidate the concepts of primary and secondary mechanical instabilities, Fig. 8 showcases the progression of cortical folding from gestational week 24 to 30 in a partial coronal section of the developing brain along with the corresponding entire fetal brain surfaces and coronal sections. Throughout the examined period, the simulation results well capture the developmental trajectory and cortical folding patterns observed in the MRI sections. Until gestational week 26, the initial brain surface appears relatively smooth, while the deeper subcortical layer undergoes isotropic expansion, particularly the outer subventricular zone (OSVZ), as indicated by the color gradient. Conversely, as neuronal density in the cortex increases, a more circumferential expansion becomes evident compared to radial expansion. Consequently, by gestational week 27, undulations begin to appear on the outer brain surface, signifying the emergence of the first or primary mechanical instability due to critical compressive stresses. By gestational week 28, the sulci continue to deepen rapidly, consistent with observations in the real human brain. Notably, sulcus 3, as introduced in Fig. 7, ceases to deepen and instead becomes shallower, forming a period-doubling pattern after week 29. This behavior is attributed to the occurrence of a secondary mechanical instability^14,55,56^.

### Spatial and temporal type-specific density distributions

In this section, we explore the temporal and spatial evolution of the cell types under consideration from gestational week 5 to around gestational week 29. As outlined in Sect. “Model parameters”, we initially set the radius of the simulation domain to 2 mm, which does not directly correspond to the radius of the studied part of the fetal brain at gestational week 5. While this assumption is essential for conserving numerical stability and consistency, by scaling, we can still accurately evaluate the evolution of cellular processes. To this end, we scale the radius of the simulation results, as discussed in Sect. “Comparative visualization: MRI sections vs. simulation results”. Simultaneously, we initiate biological processes from gestational week 5. Consequently, the simulation results spanning gestational weeks 5 to before 24 do not accurately reflect genuine geometric changes and are presented without units.

**Fig. 9:**
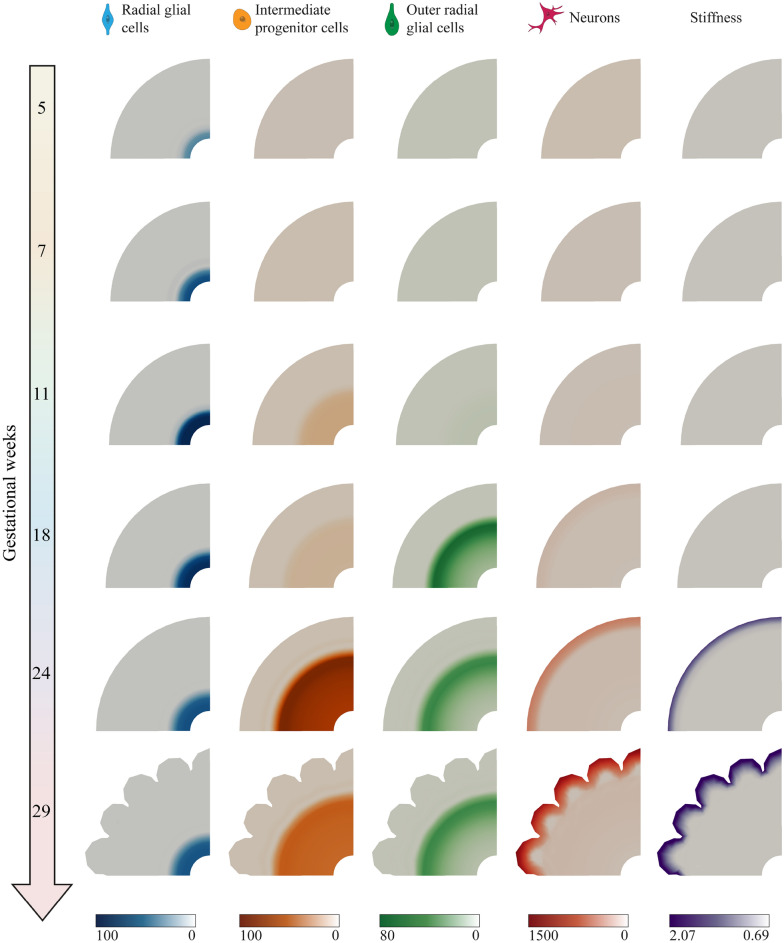
Illustrative progression of type-specific cell density, stiffness evolution, and cortical folding patterns across gestational weeks. Evolving distribution of four different cell types in the human brain, radial glial cells (RGCs), intermediate progenitor cells (IPCs), outer radial glial cells (ORGCs), and neurons alongside stiffness from gestational weeks 5 through 29. The results were obtained under varying stiffness conditions, utilizing the parameters detailed in Table 2. The following software was used to generate this figure: Adobe Illustrator (V28.7.1), and ParaView (V5.11).

**Fig. 10:**
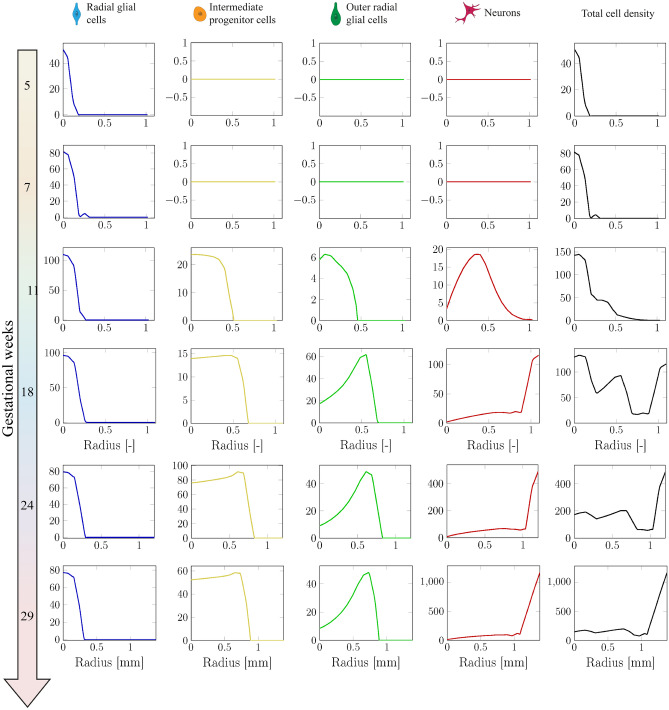
Temporal evolution of the type-specific and total cell density across a line in the radial direction from the ventricular surface to the outer cortical surface for the numerical simulations results in Fig. 9. The origin on the x-axis (0 point) corresponds to the ventricular surface rather than the center of the simulation domain.

Figure 9 presents the temporal evolution of type-specific cell density and cortical folding patterns throughout gestational weeks, focusing on the four different cell types relevant for human brain development, as introduced in Sect. “Biological background”. Corresponding temporal evolution curves are displayed in Fig. 10. The early neurogenesis stage (between gestational weeks 5 and 7) is characterized by the even proliferation of radial glial cells (RGCs) around the cerebral ventricle, leading to an increase in the thickness of the ventricular zone (VZ), thereby enhancing its capacity to generate more cells. Between gestational weeks 7 and 11, RGCs reach their peak density and begin asymmetric division, producing intermediate progenitor cells (IPCs) that transport regularly to fill the space between the VZ and outer subventricular zone (OSVZ). In our model, each IPC divides to generate another IPC and a pair of neurons. Initially, these neurons migrate slowly, which can be attributed to the limited number of glial fibers, as outer radial glial cells (ORGCs) have not yet been produced. After gestational week 11, the number of RGCs remains constant, but they undergo asymmetric division to produce ORGCs instead of IPCs. These ORGCs rapidly translocate to the OSVZ and exhibit mitotic small translocation division behavior, increasing OSVZ thickness consistently. This unique division behavior enhances the capacity of the OSVZ, providing more space for producing IPCs and neurons. By gestational week 18, the OSVZ displays a high density of ORGCs, while IPCs remain dissipated uniformly in the main space between the VZ and OSVZ, and neurons populate the cortex. Notably, the geometrical radius increases due to isotropic growth in deeper layers, in particular in the VZ and the OSVZ, while the thickness of the ISVZ remains constant over time. After gestational week 24, RGC and ORGC densities decrease due to increased VZ and OSVZ thickness, respectively, while IPC density notably rises, as they are sourced from two origins: ORGCs and self-amplification. Until gestational week 24, cortical folds have not yet emerged, despite the cortex being densely populated with neurons, indicating that tangential forces have not reached the critical buckling threshold. Towards the end of the simulation, RGC and ORGC densities remain constant, with no further increases in deeper layer thickness. Conversely, IPC density declines significantly as they undergo asymmetric division to produce cortical neurons. As a result, cortical neuron density increases significantly. Neurons migrate directly towards the brain surface by using the fibers of ORGCs. They populate the upper cortical layers, which initiates connectivity formation. This process contributes to the emergence of folds, as tangential forces approach the instability point.

The column on the far right in Fig. 9 illustrates how stiffness evolves throughout gestation. In the early and mid-gestational stages, the density of cortical cells, particularly neurons, remains below the minimal threshold $c^{\mu }_{\text{min}}$, resulting in uniform tissue stiffness corresponding to a shear modulus of $\mu _{\text{s}} = {0.69}\,{\text{kPa}}$. By gestational week 24, neuron density in the cortex surpasses this threshold, leading to a gradual increase in cortical stiffness as neuron density rises, eventually reaching the maximum value corresponding to the shear modulus of the fully developed brain ${\mu }_{\infty } = {2.07}\,{\text{kPa}}$.

**Fig. 11:**
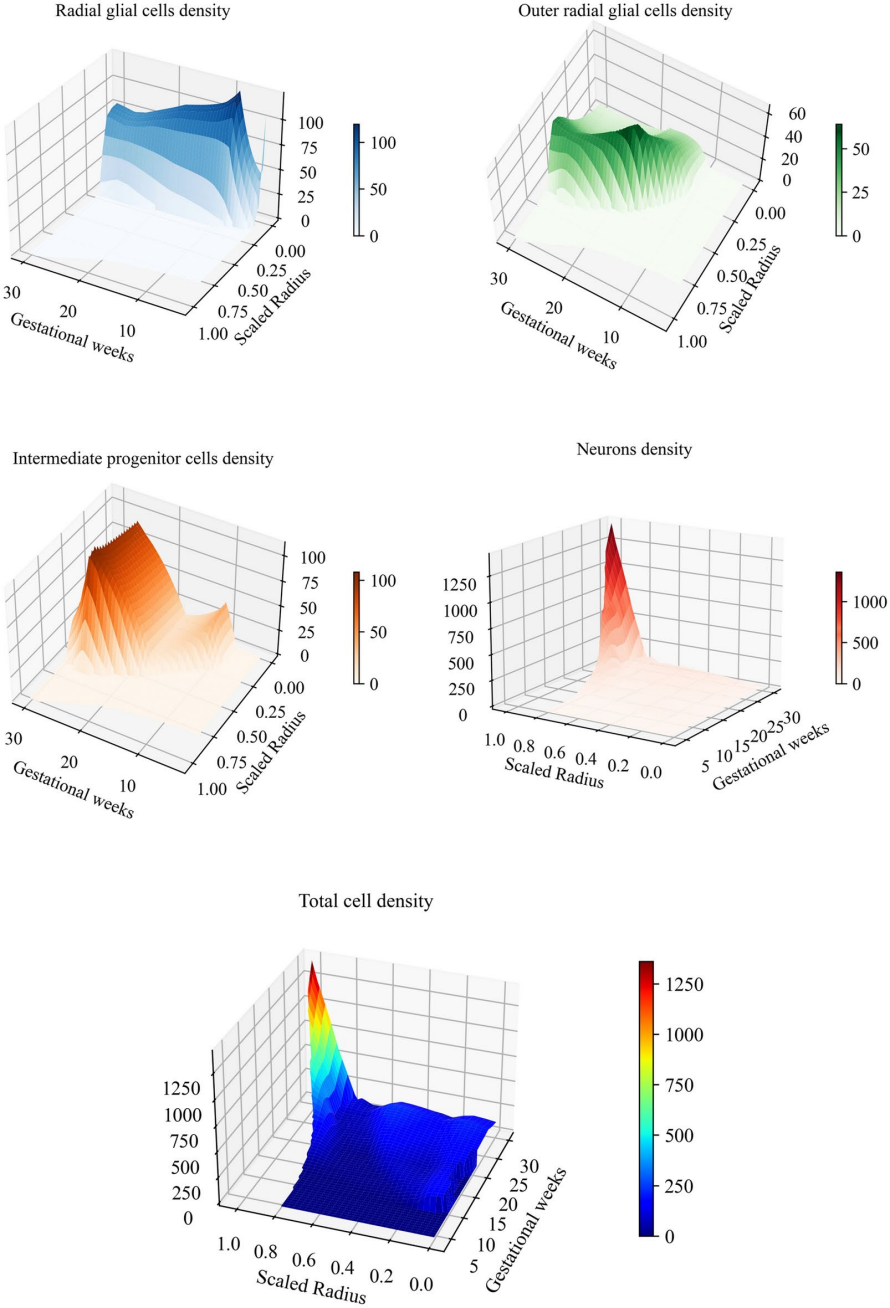
Spatio-temporal distribution of the cell density obtained from numerical simulation for various cell types in the developing brain: radial glial cells (RGCs, upper left), intermediate progenitor cells (IPCs, middle left), outer radial glial cells (ORGCs, upper right), and neurons (middle right), along with the total cell density (bottom). Each graph illustrates changes in cell density over time and radial distance from the ventricular surface, with unique color gradients representing the density levels of each cell type. Please be aware that we use a different axis orientation for each graph to highlight all specific details. This figure was created using a Python script (V3.11.6) with the Matplotlib package (V3.8.0)^59^.

To capture further details regarding the evolution of the cell density, we include Fig. 11, which demonstrates 3D curves of the spatial-temporal evolution of the type-specific cell density obtained from the numerical simulation using the parameters summarized in Table 2. Here, the x-axis denotes the progression of gestational weeks, while the y-axis represents the radial distance from the ventricular surface. Once more, we do not account for the actual alteration in the radius of the real human brain, owing to the disparity in the metric before and after gestational week 24. To prevent any confusion, we scale the radius relative to the maximum radius within the domain. Please be aware that we use a different axis orientation for each graph to highlight all specific details.

The radial glial cells’ graph (upper left) illustrates a swift increase in cell density from gestational weeks 5 to 10, reaching its peak at week 9. Subsequently, the density gradually and almost linearly decreases until around gestational week 24. The increase in the radius of the ventricular zone during this period substantiates our earlier allegation regarding this phenomenon. Between gestational weeks 24 and 30, the cell density remains constant, as the expansion of deeper subcortical layers, including the ventricular zone, halts during this period.

The outer radial glial cells’ graph (upper right) shows the absence of these cells in the first trimester of the gestational life. By the onset of the second trimester (gestational week 11), the density of ORGCs begins to rise, reaching its major peak around gestational week 20, before gradually declining. This decline is not solely attributed to the expansion of the subcortical layer due to growth, but also to the influence of mitotic somal translocation (MST). As we observe, ORGCs are rarely found in the VZ and ISVZ, with their presence primarily restricted to the translocation of these cells from their original birthplace to the OSVZ between gestational weeks 11 and 18.

In contrast, the graph illustrating intermediate progenitor cells (middle left) exhibits two peaks since they originate from two distinct sources besides self-amplification, which are RGCs and ORGCs. At the onset of the second division phase (gestational week 7), IPCs are generated basically from RGCs, and the newly formed IPCs subsequently divide to produce additional IPCs and neurons. This process continues until gestational week 11, during which the first (minor) peak occurs. Subsequently, between gestational week 11 and 18, the primary source of IPCs, namely RGCs, transitions to produce ORGCs. Consequently, the density of IPCs undergoes a notable decrease since they are currently produced exclusively through self-amplification. Additionally, the distribution of IPCs within larger space, in particular the SVZ, contributes to a reduction in their density. Once again, at the beginning of the fourth division phase, the density undergoes a notable increase, reaching its second (major) peak. The higher value of the second peak compared to the first one is directly attributed to the greater abundance of IPCs at the beginning of the fourth phase compared to the second phase. Hence, the cells intensively produce from one source, namely ORGCs, besides self-amplification. After gestational week 24, the density undergoes a dramatic decrease, as IPCs switch to produce cortical neurons exclusively. Unlike ORGCs, the density of IPCs shows a uniform distribution throughout the space between the VZ and the SVZ across gestational weeks.

Finally, the graph depicting neurons (lower right) appears markedly different. Up until gestational week 20, the number of neurons produced is limited. However, thereafter, it experiences a sudden and dramatic increase until reaching its maximum peak at gestational week 30. The primary cause of this wave is attributed to the substantial batch of neurons generated by IPCs division during the fifth division phase. Intriguingly, this significant extension in domain radius coincides with the rise in neuron density.

The spatio-temporal distribution of the total cell density, which is the sum over all the different cell types, agrees well with previous observation in the human fetal brain^35^.

### Cellular deconvolution through a multi-field computational model

Uncovering the underlying cellular components of specific tissue regions, a process known as cellular deconvolution, poses one of the most challenging tasks in analyzing human brain data obtained during the developmental stages^57^. This challenge stems from limitations of computational methodologies, coupled with the complexity and high costs associated with the techniques required for this task, i.e., gene expression analyses^58^. Consequently, a computational approach based on the multi-field model presented in this study may be an alternative solution to characterize cellular heterogeneity and gain a deeper understanding of the composition of complex biological samples.

**Fig. 12:**
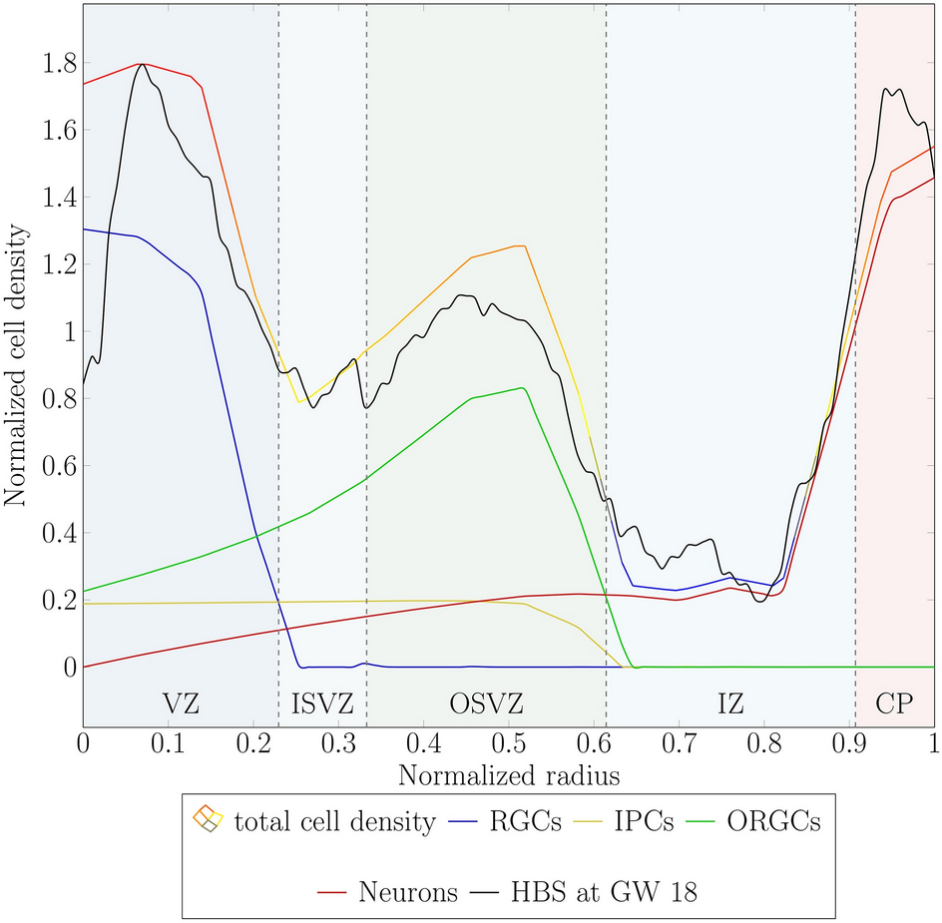
Radial cell density distribution at gestational week 18. Cell type deconvolution for the total cell density with its four cellular components, radial glial cells (RGCs), intermediate progenitor cells (IPCs), outer radial glial cells (ORGCs), and neurons, across various brain zones: ventricular zone (VZ), inner subventricular zone (ISVZ), outer subventricular zone (OSVZ), intermediate zone (IZ), and cortical plate (CP), along the normalized radius of the brain. The data is benchmarked against a human brain section (HBS) at gestational week 18. The curves of the simulation results are normalized with respect to the average of the total cell density. Similarly, the HBS data is normalized with respect to its average value.

In Sect. “Model parameters”, we calibrated the model parameters using histological human brain sections obtained at gestational week 18. The trends observed in the total cell density curve closely match that of the human brain sections. Still, the specific cellular components contributing to the total density in each germinal zone have not been investigated. Therefore, we present Fig. 12, which illustrates the cellular deconvolution of the total cell density at gestational week 18 into its fundamental cellular components: radial glial cells (RGCs), intermediate progenitor cells (IPCs), outer radial glial cells (ORGCs), and neurons. Once again, to facilitate comparison, we normalized the curves on the x and y axes with respect to the maximum and average values, respectively (for further details, see Sect. “Model parameters”). As expected, in the first, innermost proliferation zone, i.e., the ventricular zone (VZ), RGCs constitute the primary cellular component, although they are not the sole occupants. Additionally, IPCs and ORGCs, which originate from RGCs, are also present in the VZ. Furthermore, migrating neurons produced by IPCs are observed in this region. The higher density of the cells in the VZ compared with other regions contributes to a massive radial extension of this zone during gestational week 18. The inner subventricular zone (ISVZ) serves as a transit zone for ORGCs translocating to the outer subventricular zone (OSVZ) and neurons migrating towards the cortex. Consequently, it does not exhibit a high cell density. In the second proliferation zone, the OSVZ, the dominant cell type is ORGCs. However, both IPCs and migrating neurons are also present in this zone. In contrast, the intermediate zone (IZ) does not host any progenitor cell types. Instead, it is regarded as a transit area for migrated neurons. Finally, the cortex as expected is fully populated by neurons.

### Influence of model parameters on final folding patterns

In this section, we investigate the influence of certain modeling choices on the final folding pattern, i.e., (i) whether the cortical stiffness changes during brain development due to the increasing cell density and connectivity (varying stiffness) or whether it stays constant (constant stiffness), (ii) different ratios of stiffness and (iii) different ratios of growth betwen the cortex and inner layers. For each stiffness case, we use 25 sets of considered parameters. These sets include stiffness ratios $\beta _{\mu }$ ranging from 1 to 5, and growth ratios $\beta _{\kappa }$ ranging from 1 to 3, in increments of 0.5.

**Fig. 13:**
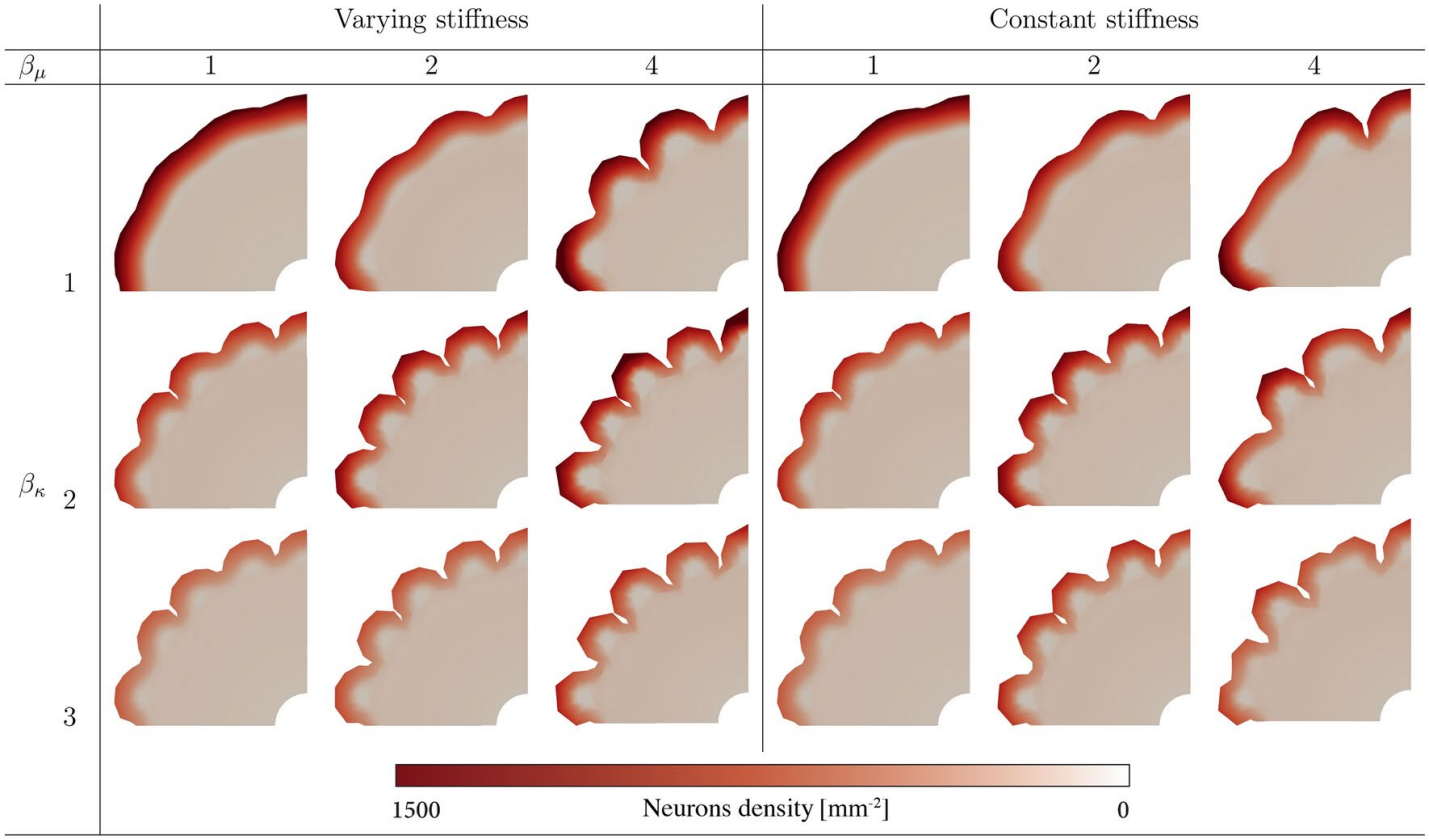
Final folding patterns at approximately gestational week 32 for different parameter sets for the variable (left) and constant (right) cortical stiffness cases. Two of the most influential factors are varied, the stiffness ratio $\beta _{\mu }$ (increasing from 1 to 5 from left to right), and the growth ratio $\beta _{\kappa }$ (increasing from 1 to 3 from top to bottom).

Figure 13 shows the emerging folding pattern at approximately gestational week 34 for the variable (left) and constant (right) stiffness cases. In general, the complexity of the folds increases from top to bottom and from left to right, corresponding to increasing values of both the stiffness and growth ratio. For the varying cortical stiffness case and parameter set $\{ \beta _{\mu }= 1, \beta _{\kappa } = 1 \}$, the brain surface appears almost smooth. The difference in stiffness and growth is not large enough to induce folding. However, as the stiffness ratio increases, the sulci deepen and the folding patterns become more irregular. For higher growth ratios, the wavelength of folds, i.e., the distance between neighboring sulci, decreases. For the constant stiffness case, the distance between sulci is generally larger, especially for higher stiffness ratios. In addition, some patterns exhibit higher complexity than for the varying stiffness case. We also observe that the wavelength of folds generally increases with an increasing stiffness ratio, which is consistent with previous studies and analytical predictions^9^. In turn, the wavelength of folds decreases for increasing growth ratios.

**Fig. 14:**
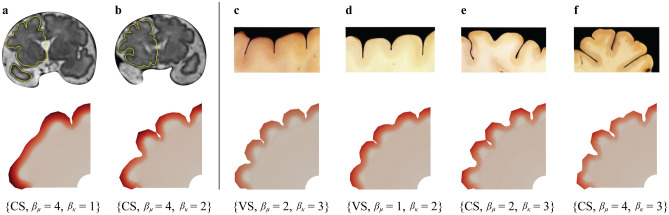
Representative folding patterns observed in the real human brain and corresponding example of numerical simulations. Panels (a) and (b) showcase the folding patterns from magnetic resonance imaging coronal sections of a fetal human brain at 34 weeks of gestation. Panel (c) shows a periodic wrinkling pattern. Panels (d) to (f) show different types of period-doubling patterns in mammalian brains^56^. The parameter set that produces the simulation results is clarified for each result, where ’CS’ indicates constant stiffness and ’VS’ indicates varying stiffness.

Figure 14 shows a comparison of representative folding patterns in the simulations and corresponding morphologies in human and mammalian brains.

For instance, the result for the constant stiffness case and parameter set $\{ \beta _{\mu }= 4, \beta _{\kappa } = 1 \}$ features one deep sulcus next to a large flat gyrus, mirroring characteristics observed in magnetic resonance (MR) images of fetal brains, as shown in Fig. 14 a. Furthermore, the pattern generated by the parameter set $\{ \beta _{\mu }= 2, \beta _{\kappa } = 2 \}$ exhibits a distinctive morphology not seen for the varying stiffness case that closely matches the magnetic resonance imaging (MRI) section in Fig. 14 b. For the parameter set $\{ \beta _{\mu }= 2, \beta _{\kappa } = 3 \}$ of the varying stiffness case, the simulation result exhibits characteristic periodic wrinkling patterns, as shown in Fig. 14 c. For the parameter set $\{ \beta _{\mu }= 1, \beta _{\kappa } = 2 \}$, the resulting pattern displays period-doubling patterns, where every second amplitude is larger than the ones in between^5^, which one can also see in real brains, as depicted in Fig. 14 d. Additionally, a different type of period-doubling pattern, similar to those in Fig. 14 e, is observed for the parameter set $\{ \beta _{\mu }= 4, \beta _{\kappa } =3 \}$. For the constant stiffness case, three types of period-doubling bifurcations are generated. The first type is similar to the one in the varying stiffness case for the parameter set $\{ \beta _{\mu }= 1, \beta _{\kappa } =2 \}$. The second type occurs with the parameter set $\{ \beta _{\mu }= 2, \beta _{\kappa } = 3 \}$, which closely mirrors the pattern depicted in Fig. 14 e. Finally, the third type occurs when growth and stiffness ratios satisfy $\{ \beta _{\mu }= 4, \beta _{\kappa } = 3 \}$, and resembles the pattern observed in the actual mammalian brain, as shown in Fig. 14 f.

**Fig. 15:**
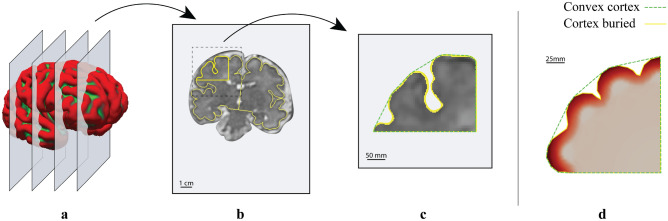
Determination of local gyrification index (lGI) for magnetic resonance (MR) images of the human fetal brain at 34 weeks of gestation (a - c) and corresponding simulation results (d). The initial stage for processing the magnetic resonance imaging (MRI) scans involves creating a series of coronal slices that traverse the 3D structure of the brain from front (anterior) to back (posterior) (a). One such coronal section is accentuated, with the cerebral cortex demarcated in yellow (b). This image is then segmented into discrete areas reflecting the simulation’s layout, enabling the calculation of the lGI for these specific zones (c).

To determine if a constant or variable cell-density-dependent cortical stiffness more accurately reflects the situation in the human brain, we assess the local gyrification index (lGI) as a criterion to compare simulation results with MR images of the human fetal brain at 34 weeks of gestation. The local gyrification index, a metric indicating the complexity of cortical folds, is calculated as the ratio between the whole outer contour of the cortex and the convex (or outer visible) hull. We use a custom-written tool, implemented in Python, named *FetoMorph* to compute this value from both simulation results and MR images, as illustrated in Fig. 15. For processing the MR images, the initial stage involves creating a series of coronal slices that traverse the 3D reconstructed structure of the brain from anterior to posterior. Then, each section is segmented into discrete areas that reflect the simulation layout, as described in Sect. “Geometry and discretization in space”. The final step involves calculating the lGI for those specific areas.

**Fig. 16:**
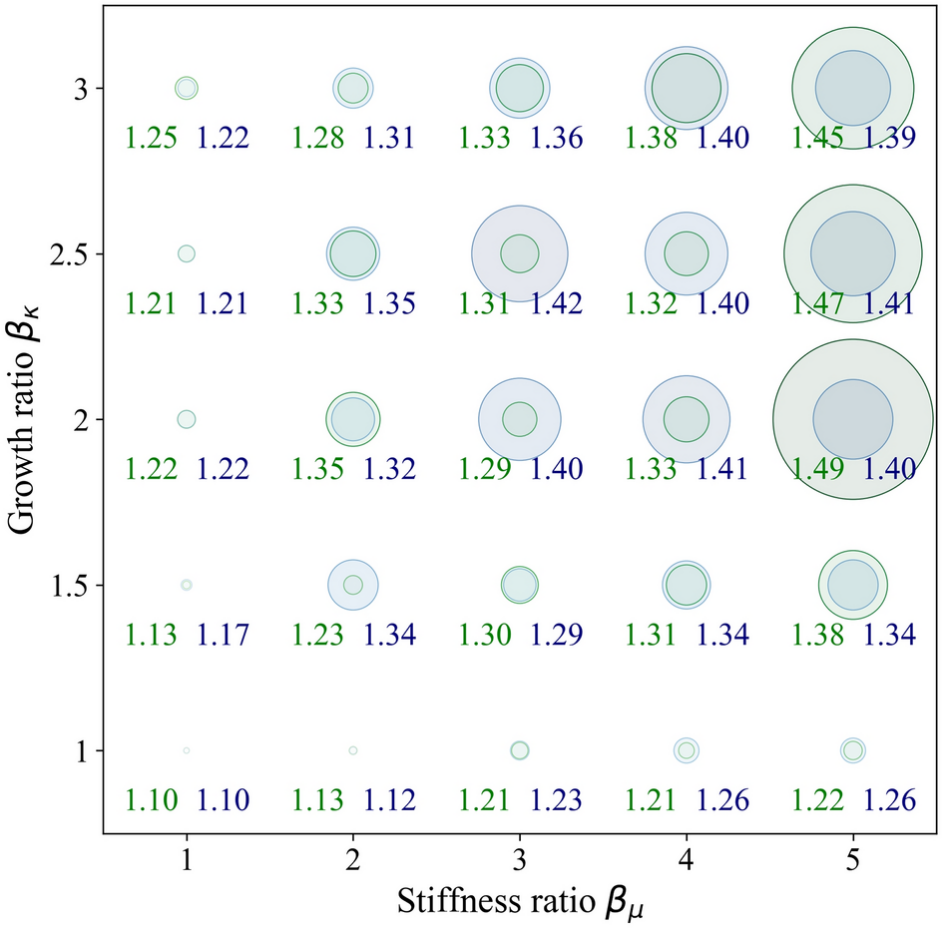
Bubble chart illustrating and contrasting the local gyrification index (lGI) values for cases with variable cortical stiffness (blue) and constant cortical stiffness (green), with the growth ratio ranging from 1 to 3, and the stiffness ratio from 1 to 5. The size of each bubble is exponentially amplified to reflect the magnitude of the lGI. Numerical values under each bubble indicate the lGI value for the cases of constant and variable cortical stiffness in green and blue, respectively.

Figure 16 illustrates the measured lGI values from simulation results for scenarios with variable (blue) and constant (green) cortical stiffness, as shown in Fig. 13. Generally, no significant differences are observed between the two scenarios when the growth ratio is below 1.5 or the stiffness ratio is less than 2. However, as both the stiffness and growth ratios increase, the lGI values in the variable cortical stiffness scenario tend to meet or exceed those in the constant scenario, especially when $3 \le \beta _{\mu } < 5$ and $2 \le \beta _{\kappa }$. Surprisingly, when the stiffness ratio surpasses 5, the lGI values escalate significantly for the constant compared to the variable cortical stiffness scenario. This shift can primarily be attributed to the emergence of period-doubling patterns at higher stiffness and growth ratios, which augments the outer surface area of the human brain. These findings align with our prior observations that simulations under constant stiffness conditions tend to display more intricate patterns at higher stiffness ratios^14,35^. It seems that reduced stiffness variation between the cortex and subcortex during development in the variable stiffness scenario promotes the evolution of complex folds at lower stiffness ratios, i.e., $\beta _{\mu } < 5$. Conversely, the constant stiffness scenario induces more elaborate folds at higher stiffness ratios, i.e., $\beta _{\mu }>5$

.

**Fig. 17:**
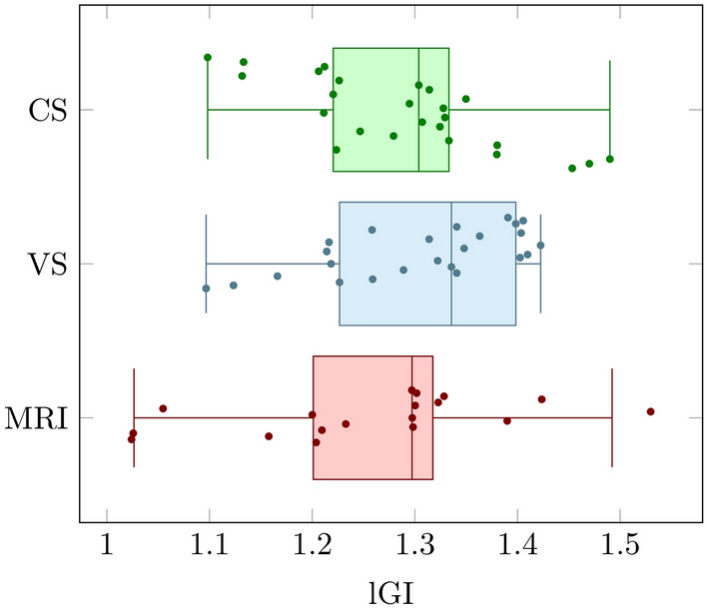
Average local gyrification index (lGI) obtained from simulation results, considering both constant (CS) and variable cortical stiffness cases (VS), compared to the values of magnetic resonance imaging (MRI) coronal sections of the real human fetal brain.

Our previous works had suggested that cortical stiffness tends to vary rather than remain constant during brain development^13,35^. In this work, we follow a similar approach as previously, performing a statistical analysis of simulation results and real human brain data, to assess whether the constant or variable cortical stiffness case for the now extended multi-field computational model better matches the data of the real human brain. For this analysis, we use a sample size of $n = 25$ for each simulation case, as shown in Fig. 16, and $n = 18$ for the MRI coronal sections. Figure 17 highlights the average lGI values with confidence intervals obtained from the simulation results with constant cortical stiffness (CS) and variable cortical stiffness (VS), besides the data acquired from MRI sections. We observe that the mean values for both stiffness scenarios are quite similar, with $1.289 \pm 0.099$ for constant stiffness and $1.306 \pm 0.0935$ for varying stiffness. Furthermore, the lGI values from the MRI sections show $1.255 \pm 0.13$, which closely matches the simulated values. To further evaluate if the differences between the lGI values obtained for the different simulation cases and the MR images are significant, we apply the pairwise t-tests with Bonferroni correction, a method used for multiple comparisons. The results indicate that there are no statistically significant differences between the MRI section measurements and both the constant and varying stiffness scenarios, with p-values greater than the adjusted alpha level of 0.0167 for each pairwise comparison. Thus, based on the folding pattern alone, it is not possible to draw a conclusion towards whether the constant or varying cortical stiffness case is more realistic.

## Conclusion

We have presented a multi-field computational model that integrates the biological behavior of different cell types during human brain development and mechanical deformation and growth. We considered the lineage connections between different cell types and their distinct behaviors regarding migration, diffusion, and even unique phenomena like mitotic small translocation in outer radial glial cells. This has allowed us to investigate how these processes impact cortical folding patterns. The model effectively tracks the visual changes observed in magnetic resonance (MR) images of the human fetal brain between gestational weeks 24 and 30. Additionally, it accurately captures the spatial and temporal distribution of different cell type densities, while providing a mechanical explanation for the phenomena observed during development. Moreover, we demonstrate that the model could be useful to uncover the underlying cellular components of histological sections of the human brain. By carefully adjusting the model parameters, it successfully replicates various folding patterns observed in the real human brain, including periodic wrinkling, different types of periodic-doubling patterns, and even unique patterns seen in coronal MR images of the human brain. In line with our previous work, we investigated two scenarios concerning cortical stiffness, one with constant stiffness value and the other with stiffness varying according to the increasing cell density. Statistical comparison with MR images of the human brain at gestational week 34, in terms of the local gyrification index (lGI), did not conclusively determine which scenario better represents the situation in the real human brain.

The presented model provides important insights into the interplay between cellular processes and folding patterns. It can help uncover the roles of different cell types under both physiological and pathological conditions. With the possibility of adjusting the model parameters, we can correlate microscopic observations from histologically stained sections with macroscopic changes seen in MR images. Consequently, the model can assist clinicians in identifying the cellular mechanisms behind cortical malformations, such as lissencephaly and polymicrogyria, for better diagnosis and treatment strategies.

Although our model demonstrates good agreement with the MR images, it requires further calibration and optimization of its input parameters - ideally by considering cellular and morphological data simultaneously. This step necessitates comprehensive data sets, which we are currently generating in collaboration with our partners. Importantly, our model is not limited to human brain development; it can also be applied to other species with convoluted brains, such as ferrets. Using histologically stained sections of ferrets to calibrate model parameters, which are more readily obtainable than those of humans, presents a promising avenue for extending our model.

## Supplementary Information

Supplementary Information.

## Data Availability

Both dHCP fetal and neonatal MRI as well as surface data that support the findings of this study are publicly released at https://biomedia.github.io/dHCP-release-notes/. The simulation results and all other data used in this study are available from the corresponding author upon reasonable request.
