# Supplementary material for: Exploring the role of different cell types on cortical folding in the developing human brain through computational modeling
Source: Sci Rep. 2024 Oct 30;14:26103. doi: 10.1038/s41598-024-75952-7 (PMC11525573; doi:10.1038/s41598-024-75952-7)
Supplement: Supplementary file 1 — Supplementary Information. [file 41598_2024_75952_MOESM1_ESM.pdf]

Supplementary Information for: Exploring the role of different cell types on cortical folding in the developing human brain through computational modeling.

Mohammad Saeed Zarzor[1*], Qiang Ma[2], Median Almurey[1], Bernhard Kainz[2,3], and Silvia Budday[1*]

[1]Institute of Continuum Mechanics and Biomechanics, Friedrich-Alexander-Universität Erlangen-Nürnberg, Erlangen, 91058, Germany
[2]Department of Computing, Imperial College London, London, SW7 2AZ, UK
[3]Erlangen Graduate School in Advanced Optical Technologies,Friedrich-Alexander-Universität Erlangen-Nürnberg, Erlangen, 91052, Germany
[*]saeed.zarzor@fau.de; silvia.budday@fau.de

Supplementary material

Supplementary Table 1: The divsion ratio between cells $G_{\bullet}(\circ, \mathrm{P})$ throughout various division phases

	G_{RG}	G_{IP}	G_{ORG}	G_{N}
P1				
RG	2	0	0	0
IP	0	0	0	0
ORG	0	0	0	0
N	0	0	0	0
P2				
RG	1	1	0	0
IP	0	1	0	2
ORG	0	0	0	0
N	0	0	0	0
P3				
RG	1	0	1	0
IP	0	1	0	2
ORG	0	0	1	0
N	0	0	0	0
P4				
RG	1	0	0	0
IP	0	1	0	2
ORG	0	1	1	0
N	0	0	0	0
P5				
RG	1	0	0	0
IP	0	0	0	4
ORG	0	0	1	0
N	0	0	0	0

Calculating the source terms of the model in Equation 14 for each division phase, based on the lineage tree illustrated in Figure 5.

The first phase:

$$f_{\mathrm{RG}} = G_{\mathrm{RG}}(\mathrm{RG}, \mathrm{P1})\, c_{\mathrm{RG}} = 2\, c_{\mathrm{RG}}$$

The second phase:

$$\begin{aligned} f_{\mathrm{RG}} &= G_{\mathrm{RG}}(\mathrm{RG}, \mathrm{P2})\, c_{\mathrm{RG}} = 1\, c_{\mathrm{RG}} \\ f_{\mathrm{IP}} &= G_{\mathrm{IP}}(\mathrm{RG}, \mathrm{P2})\, c_{\mathrm{RG}} + G_{\mathrm{IP}}(\mathrm{IP}, \mathrm{P2}) c_{\mathrm{IP}} = 1\, c_{\mathrm{RG}} + 1\, c_{\mathrm{IP}} \\ f_{\mathrm{N}} &= G_{\mathrm{N}}(\mathrm{IP}, \mathrm{P2})\, c_{\mathrm{IP}} = 2\, c_{\mathrm{IP}} \end{aligned}$$

The third phase:

$$\begin{aligned} f_{\mathrm{RG}} &= G_{\mathrm{RG}}(\mathrm{RG}, \mathrm{P3})\, c_{\mathrm{RG}} = 1\, c_{\mathrm{RG}} \\ f_{\mathrm{ORG}} &= G_{\mathrm{ORG}}(\mathrm{RG}, \mathrm{P3})\, c_{\mathrm{RG}} + G_{\mathrm{ORG}}(\mathrm{ORG}, \mathrm{P3})\, c_{\mathrm{ORG}} = 1\, c_{\mathrm{RG}} + 1\, c_{\mathrm{ORG}} \\ f_{\mathrm{IP}} &= G_{\mathrm{IP}}(\mathrm{IP}, \mathrm{P3})\, c_{\mathrm{IP}} = 1\, c_{\mathrm{IP}} \\ f_{\mathrm{N}} &= G_{\mathrm{N}}(\mathrm{IP}, \mathrm{P3})\, c_{\mathrm{IP}} = 2\, c_{\mathrm{IP}} \end{aligned}$$

The fourth phase:

$$\begin{aligned} f_{\mathrm{ORG}} &= G_{\mathrm{ORG}}(\mathrm{ORG}, \mathrm{P4})\, c_{\mathrm{ORG}} = 1\, c_{\mathrm{ORG}} \\ f_{\mathrm{IP}} &= G_{\mathrm{IP}}(\mathrm{IP}, \mathrm{P4})\, c_{\mathrm{IP}} + G_{\mathrm{IP}}(\mathrm{ORG}, \mathrm{P4})\, c_{\mathrm{ORG}} = 1\, c_{\mathrm{IP}} + 1\, c_{\mathrm{ORG}} \\ f_{\mathrm{N}} &= G_{\mathrm{N}}(\mathrm{IP}, \mathrm{P4})\, c_{\mathrm{IP}} = 2\, c_{\mathrm{IP}} \end{aligned}$$

The fifth phase:

$$f_{\mathrm{N}} = G_{\mathrm{N}}(\mathrm{IP}, \mathrm{P5})\, c_{\mathrm{IP}} = 4\, c_{\mathrm{IP}}$$
